# Sexually dimorphic DNA methylation and gene expression patterns in human first trimester placenta

**DOI:** 10.1186/s13293-024-00629-9

**Published:** 2024-08-16

**Authors:** Tania L. Gonzalez, Bryn E. Willson, Erica T. Wang, Kent D. Taylor, Allynson Novoa, Akhila Swarna, Juanita C. Ortiz, Gianna J. Zeno, Caroline A. Jefferies, Kate Lawrenson, Jerome I. Rotter, Yii-Der Ida Chen, John Williams, Jinrui Cui, Mark O. Goodarzi, Margareta D. Pisarska

**Affiliations:** 1 Department of Obstetrics and Gynecology, Cedars-Sinai Medical Center, 8635 West 3rd Street, Suite 160, Los Angeles, CA 90048 USA; 2 David Geffen School of Medicine, University of California, Los Angeles, Los Angeles, CA USA; 3 The Institute for Translational Genomics and Population Sciences, Department of Pediatrics, The Lundquist Institute for Biomedical Innovation at Harbor-UCLA Medical Center, Torrance, CA USA; 4 Department of Biomedical Sciences, Cedars-Sinai Medical Center, Los Angeles, CA USA; 5 Division of Rheumatology, Department of Medicine, Kao Autoimmune Institute, Cedars-Sinai Medical Center, Los Angeles, CA USA; 6 Samuel Oschin Comprehensive Cancer Institute, Cedars-Sinai Medical Center, Los Angeles, CA USA; 7 Division of Endocrinology, Diabetes and Metabolism, Department of Medicine, Cedars-Sinai Medical Center, Los Angeles, CA USA

**Keywords:** Placenta, Early pregnancy, Healthy pregnancy, Fetal sex, DNA methylation, Total RNA-seq, Sexual dimorphism

## Abstract

**Background:**

Fetal sex and placental development impact pregnancy outcomes and fetal–maternal health, but the critical timepoint of placenta establishment in first trimester is understudied in human pregnancies.

**Methods:**

Pregnant subjects were recruited in late first trimester (weeks 10–14) at time of chorionic villus sampling, a prenatal diagnostic test. Leftover placenta tissue was collected and stored until birth outcomes were known, then DNA and RNA were isolated from singleton, normal karyotype pregnancies resulting in live births. DNA methylation was measured with the Illumina Infinium MethylationEPIC BeadChip array (n = 56). Differential methylation analysis compared 25 females versus 31 males using a generalized linear model on 743,461 autosomal probes. Gene expression sex differences were analyzed with RNA-sequencing (n = 74). An integrated analysis was performed using linear regression to correlate gene expression and DNA methylation in 51 overlapping placentas.

**Results:**

Methylation analysis identified 151 differentially methylated probes (DMPs) significant at false discovery rate < 0.05, including 89 (59%) hypermethylated in females. Probe cg17612569 (*GABPA*, *ATP5J*) was the most significant CpG site, hypermethylated in males. There were 11 differentially methylated regions affected by fetal sex, with transcription factors *ZNF300* and *ZNF311* most significantly hypermethylated in males and females, respectively. RNA-sequencing identified 152 genes significantly sexually dimorphic at false discovery rate < 0.05. The 151 DMPs were associated with 18 genes with gene downregulation (P < 0.05) in the direction of hypermethylation, including 2 genes significant at false discovery rate < 0.05 (*ZNF300* and *CUB and Sushi multiple domains 1, CSMD1*). Both genes, as well as *Family With Sequence Similarity 228 Member A (FAM228A)*, showed significant correlation between DNA methylation and sexually dimorphic gene expression, though *FAM228A* DNA methylation was less sexually dimorphic. Comparison with other sex differences studies found that cg17612569 is male-hypermethylated across gestation in placenta and in human blood up to adulthood.

**Conclusions:**

Overall, sex dimorphic differential methylation with associated differential gene expression in the first trimester placenta is small, but there remain significant genes that may be regulated through methylation leading to differences in the first trimester placenta.

**Supplementary Information:**

The online version contains supplementary material available at 10.1186/s13293-024-00629-9.

## Plain language summary

Fetal sex and placenta development affect pregnancy outcomes for both the fetus and mother throughout pregnancy, including risk of miscarriages, preterm birth, preeclampsia, and other outcomes. Epigenetics, the “overlay” of regulatory signals on DNA which affects how DNA is read, is not well understood in early pregnancy when critical placenta developments are happening that affect the rest of pregnancy. Here, we use leftover placenta biopsy samples (n = 56) donated by Cedars-Sinai patients with informed consent to learn about first trimester human placenta DNA methylation differences due to fetal sex. Out of the total 743,461 sites analyzed, we identified 151 sites significantly affected by fetal sex after correcting p-values to reduce false positives (false discovery rate < 0.05). We also performed an analysis to look at multiple sites and identified 11 regions across the genome with significant DNA methylation changes due to fetal sex. Furthermore, because DNA methylation is a regulatory mark on DNA which typically dampens gene expression, we also compared the DNA methylation sex differences to placental RNA-sequencing gene expression analysis using the same tissue from a mostly overlapping patient group (n = 74 total sequenced, n = 51 overlap). We identify 18 genes which show both significant DNA methylation differences and gene expression changes. The most significant gene was transcription factor *ZNF300* with higher DNA methylation in males and reduced gene expression in males (and thus higher gene expression in females). This study identifies some sex differences that continue until later pregnancy and others that are unique to first trimester.

**Supplementary Information:**

The online version contains supplementary material available at 10.1186/s13293-024-00629-9.

## Highlights

- We identify sex differences in first trimester human placenta DNA methylation (n = 56) and total RNA-sequencing (n = 74), with a sample overlap of n = 51.
- At late first trimester, there are 151 differentially methylated probes (DMPs), significantly different between female and male placenta at false discovery rate < 0.05. These encompass 11 differentially methylated regions.
- The gene with placental DNA methylation most affected by fetal sex is transcription factor *ZNF300*, hypermethylated in males, and with significantly female upregulated gene expression in first trimester placenta, consistent with previous third trimester studies.
- In addition to *ZNF300*, other genes in significantly differentially methylated regions at first trimester include *GABPA/ATP5J* (hypermethylated in males); *ZNF311*, *CCDC178*, *ATP5G2*, *FOXG1* (females); *ZNF175* (males); *SGCE* (females); *C6orf47-AS1/C6orf47*, *REC8*, and *FOXA1* (females).
- We identify 18 genes with significant fetal sex-associated changes in both placenta DNA methylation and placenta gene expression. The most significant are *ZNF300*, tumor suppressor *CSMD1*, and transcription factor *ZNF311.*

**Supplementary Information:**

The online version contains supplementary material available at 10.1186/s13293-024-00629-9.

## Background

The placenta is a transient organ that facilitates the growth of the fetus by exchanging nutrients and metabolic biproducts with the maternal circulation. Responsible for the growth and wellbeing of the fetus, it is also a source of many pregnancy-related morbidities. Specifically, the placenta has been implicated in fetal growth restriction and hypertensive diseases of pregnancy [1–3]. Fetal sex is also known to impact pregnancy related morbidities [4, 5]. Male fetal sex (XY) has been associated with increased rates of preterm delivery, preterm prelabor rupture of membranes [4], as well as increased rates of gestational diabetes [6]. Male fetuses are also larger on ultrasound evaluations [7]. Given these differences in morbidity and growth profiles of pregnancies with male fetuses, further understanding is needed of gene expression and the upstream regulatory mechanisms responsible for sexually dimorphic placental phenotypes, particularly in early pregnancy.

The first trimester is a critical timepoint in placenta establishment (placentation). The invasion of trophoblast cells into the uterine wall and subsequent remodeling of spiral uterine arteries is thought to be involved in the progression of placental dysfunction including pregnancy loss, growth restriction, and hypertensive disease of pregnancy [8]. Further understanding the first trimester placenta is therefore essential in understanding, and some day preventing, pregnancy related morbidities.

We and others have found sexually dimorphic gene expression in human placentas using RNA-sequencing (RNA-seq) [9–11]. Upstream regulators of gene expression such as DNA methylation at CpG dinucleotide sites is also important to understand and can vary by organs and tissues [12]. Other studies have found sex differences in methylation patterns and gene expression in term placentas [13, 14]. Inkster et al. looked at 343 term placentas and found 162 CpG sites that were differentially methylated by sex [13]. Andrews et al. looked at 532 term placentas (overlapping Inkster’s cohort) and found 5,212 CpG sites differentially methylated by sex [14]. First trimester placenta in healthy and normal karyotype pregnancies has been more difficult to study, but it is essential to understand the methylation profile of normal placentation which impacts the rest of pregnancy. First trimester placenta tissue was collected from discarded sample after chorionic villus sampling (CVS), a prenatal diagnostic test performed in late first trimester most commonly for advanced maternal age. Our cohort is composed of singleton, normal karyotype pregnancies conceived without fertility treatments, continued until live births. The goal of the present study is to evaluate human first trimester placenta for sex differences in DNA methylation and to identify which methylation changes alter gene expression.

## Methods

### Subject recruitment and sample procurement

For this sex differences analysis, we used the 56 spontaneous subjects from the original n = 138 Spontaneous/Medically Assisted/Assisted Reproductive Technology (SMAART) DNA methylation cohort, where spontaneous refers to pregnancies conceived without fertility treatments [15]. Subjects who were already undergoing chorionic villus sampling (CVS) for medical reasons were recruited with informed consent on the day of their clinical visit. Research involvement did not affect the amount of tissue collected from the patient or other clinical decisions. Chorionic villi (first trimester placenta) tissue discarded after prenatal diagnostic testing was placed in 250 µL of *RNAlater RNA stabilizing solution* (QIAGEN) and stored at − 80 °C until further processing for research. Genomics DNA and total RNA were isolated from the same tissue samples with the AllPrep DNA/RNA Mini Kit (QIAGEN) and utilized for DNA methylation and total RNA-seq projects as previously described [15, 16]. All protocols were performed in accordance with the institutional review board’s guidelines at the Cedars-Sinai Medical Center under IRB Protocols Pro00006806 and Pro00008600. All pregnancies were singleton, normal karyotype, and resulted in live births. The gestational ages range at CVS was 71–99 days (mean 82 ± 6 days standard deviation). Pregnancies delivered at 39 ± 2 weeks gestation, all full term except two preterm births in the male cohort.

### Analysis of demographic data

Demographics data collected included maternal/paternal age, race, ethnicity, maternal BMI, fetal race/ethnicity, maternal conditions such as hypertension, diabetes, thyroid disease, and other medical conditions. Pregnancy outcomes evaluated included gestational age at time of CVS, crown rump length (CRL) at time of CVS, gestational age at delivery, birthweight in grams, and mode of delivery. Pregnancy complications included hypertension, diabetes, coagulation disorders, placenta previa, placenta abruption, and other placental problems. Demographics analysis was performed to compare the cohorts by fetal sex. Means and standard deviations were reported for continuous variables and proportions were reported as percentages. The student’s T-test was used for normally distributed data and Chi-square test was used to compare categorical variables. Demographics were performed for the DNA methylation cohort (n = 56) and the RNA-seq cohort (n = 74).

### DNA methylation data and filtering

The Infinium MethylationEPIC BeadChip array was filtered to 743,461 autosomal probes, including 741,145 CpG sites and 2316 CpH sites, with the following filtering steps. Unreliable or masked probes were dropped using Dr Wanding Zhou’s EPIC array manifest file “EPIC.hg38.manifest.tsv.gz” column MASK_general = TRUE [17], release 9/9/2018 available at (https://zwdzwd.github.io/InfiniumAnnotation). Non-autosomal probes were dropped (chrX, chrY, mitochondrial sites, and NA). Probes overlapping a single nucleotide polymorphism were dropped, identified using probeType = “rs”. Probe annotations such as gene context were added from the MethylationEPIC v1.0 B5 manifest (Illumina), available at (https://support.illumina.com/array/array_kits/infinium-methylationepic-beadchip-kit/downloads.html).

### Principal components analysis

For DNA methylation, principal components analysis was performed with R package *eigen* v4.2.0 for spectral decomposition of a matrix.following methods for Eigenstrat software [18, 19]. The input was an M-values matrix of all EPIC array probes (for fetal sex verification only) or 743,461 autosomal probes (filtered as described above). For RNA-sequencing, principal components analysis was performed with R packages *DESeq2* v1.4.0 (to normalize counts for sequencing depth and perform a variance stabilizing transformation), *limma* v3.56.1 (to batch correct for two sequencing runs with function *removeBatchEffect*), and base R function *prcomp* with the top 500 most variable genes [20, 21]. Scatter plots were created and combined with R packages *ggplot2, cowplot,* and *pdftools.*

### Differentially methylated probes (DMPs), regions (DMRs), and gene ontology

The Infinium MethylationEPIC BeadChip array results for 56 placentas (25 female, 31 male) at 743,461 probes were analyzed in a generalized linear model to compare female versus male methylation (M-values) without covariates, using the *limma* v3.56.0 R package function *lmFit* [21]. The P-values were adjusted for multiple comparisons using Benjamini-Hochberg’s False Discovery Rate method to generate “FDR” values [22]. Probes were significantly sexually dimorphic at FDR < 0.05, equivalent to P < 1 × 10^–5^ in this study. Enriched gene ontology (GO) terms from differentially methylated probes (DMPs) at FDR < 0.05 were identified using R package *missMethyl* v1.34.0, which accounts for the number of probes per gene and the number of probes analyzed total [23]. Additionally, probes reached a stricter “genome-wide significance” threshold if they met unadjusted P-value < 9 × 10^–8^, the threshold recommended for the EPIC BeadChip array [24]. Quantile–quantile plots were created using R package *qqman* v0.1.9 [25]. Manhattan plots were created with the most significant 5000 probes by unadjusted P-value with R packages *ggplot2*, *ggrepel*, and *cowplot*. The x-axis uses the chromosome and GRCh38 start site from the EPIC manifest and the y-axis separates probes by either − log_10_(P-value) or *limma* output “logFC” describing direction of methylation. To identify differentially methylated regions (DMRs) between female and male placentas, methylation (M-values) for 56 placentas at 743,461 probes were input into *DMRcate* v2.14.0 functions *cpg.annotate* and *dmrcate* [26, 27]. We used the default values for Gaussian kernel bandwidth for smoothed-function estimation (lambda = 1000) and scaling factor for bandwidth (C = 2).

### RNA-sequencing and sex differences analysis

We utilized our largest dataset with total RNA-seq of spontaneous and healthy first trimester human placentas, n = 74 (34 females and 40 males), available at The National Center for Biotechnology Information Gene Expression Omnibus (NCBI GEO) under accession GSE215421 [16]. The GSE215421 data includes re-sequenced samples from bulk total RNA-seq projects under accessions GSE109082 (n = 39) and GSE131874 (n = 4) [9, 28]. For each placenta sample, the RNA and DNA were isolated on the same day using the two-column kit, AllPrep DNA/RNA Mini Kit (QIAGEN), as previously described [9, 15, 16, 28, 29]. The total RNA and genomic DNA elutions were stored at − 80 °C until use for RNA-seq or the Illumina EPIC array. The counts matrix for GSE215421 was filtered to compensate for imperfect ribosomal RNA (rRNA) depletion during library preparation. Ensembl Gene IDs with gene biotype “rRNA” in the Ensembl release 91 annotations were dropped for all samples during bioinformatics analysis. Ensembl Gene IDs with zero counts across all n = 74 samples were also dropped. The final counts matrix contained 74 placentas and 55,319 genes. Differential expression analysis was performed using R package *DESeq2* v1.40.0 to fit each gene into a negative binomial generalized linear model to compare females versus males, batch adjusted for two sequencing runs [20, 30]. The resulting Wald test P-values were adjusted for multiple comparisons using the Benjamini–Hochberg False Discovery Rate (FDR) procedure [22].

### Integrated analysis comparing gene expression and DNA methylation

DMPs with sex differences at P < 0.01 were matched to corresponding genes using the gene symbols and Ensembl Transcript IDs in the EPIC manifest (GENCODE release 12), cross-referenced with the Ensembl BioMart server using R package *biomaRt* with GRCh37 and GRCh38 (Ensembl release 91) to output Ensembl Gene IDs corresponding to specific probeIDs. These Ensembl Gene IDs were used to merge RNA-seq results (n = 74 placentas) to DNA methylation results (n = 56 placentas), creating a dataset of 35,862 matches (12,560 unique probeIDs and 7737 unique genes). Using 51 placentas with data from both experiments, gene expression was correlated to DNA methylation using a generalized linear model with R version 4.3.3 function *lm* and model “gene expression ~ methylation M values”. Specifically, gene expression was defined as counts normalized for sequencing depth (“baseMeans” from *DESeq2*), transformed with log_2_(x + 1) and then corrected for batch using *limma::removeBatchEffect.*

## Results

### Demographics

**Fig. 1:**
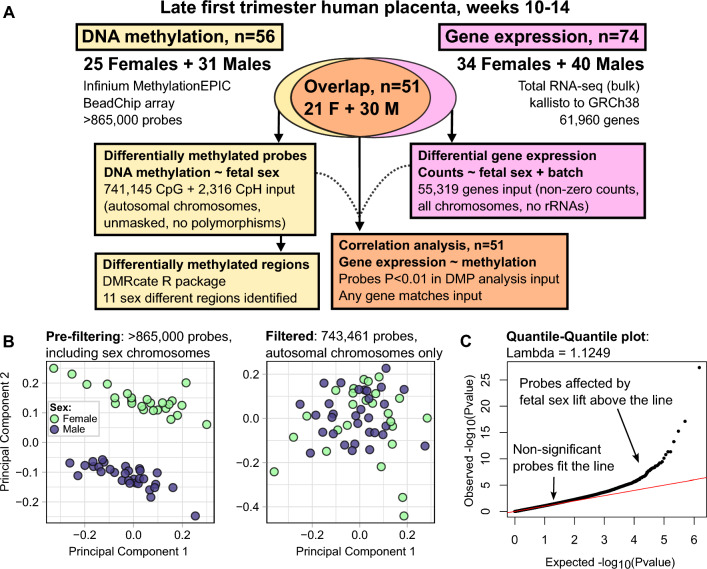
Sample cohorts and quality control. **A** Summary of the first trimester placenta cohorts and analyses. F = females, M = males. **B** Principal components analysis for the DNA methylation cohort. The pre-filtered dataset confirms fetal sex. The filtered dataset no longer shows clustering by sex. **C** Quantile–Quantile plot for the differentially methylated probes (DMP) analysis. Points are individual methylation sites (probes). Red line at P_observed_ = P_expected_

Two largely overlapping placenta cohorts were used for first trimester sex differences analyses (Fig. 1A). Demographics analyses were performed to compare each cohort by fetal sex (Table 1, Additional file 1). The methylation cohort included 56 pregnancies (25 carrying a female fetus, 31 a male fetus). The methylation cohort subjects were mostly Caucasian non-Hispanic but had no differences in maternal/paternal age, race, ethnicity, BMI, maternal medical conditions, pregnancy outcomes, or pregnancy complications (Supplemental Table S1, Table 1). The RNA-seq cohort included 74 pregnancies (34 carrying a female fetus, 40 a male fetus). RNA-seq cohort subjects were mostly Caucasian, non-Hispanic. However paternal ethnicity was more often non-Hispanic (93%) in the male fetus cohort than in the female fetus cohort (85%) (p = 0.041). No differences were noted in maternal age, race, ethnicity, BMI, maternal medical conditions, pregnancy outcomes, or pregnancy complications (Supplemental Table S2, Table 1). Principal components analysis showed that when sex chromosomes were included, samples separated by fetal sex and not by other demographics variables (Additional file 2). When sex chromosomes were excluded, fetal Hispanic samples somewhat clustered in DNA methylation data, but were still located within the larger group (Additional file 2).

**Table 1:** Brief demographics and pregnancy outcomes

	DNA methylation cohort			RNA-seq cohort		
	Females n = 25	Males n = 31	P value	Females n = 34	Males n = 40	P value
Maternal age (years)	40 ± 1.9	39 ± 2.2	0.1003	39 ± 2.0	39 ± 2.4	39 ± 2.0
Paternal age (years)	42 ± 5.8	41 ± 4.0	0.2862	41 ± 5.4	41 ± 4.6	41 ± 5.4
Maternal BMI	23 ± 3.8	22 ± 2.8	0.3908	22 ± 3.6	22 ± 2.8	0.5489
Fetal race	20 (80%) Caucasian	29 (95%) Caucasian	0.398	29 (85%) Caucasian	39 (98%) Caucasian	0.055
Fetal ethnicity	18 (72%) non-Hispanic	27 (87%) non-Hispanic	0.157	24 (71%)	35 (88%)	0.071
Gestational age at CVS (days)	82 ± 7.3	82 ± 5.5	0.8851	84 ± 6.4	82 ± 5.9	0.2350
Gestational age at delivery	276 ± 7.5	272 ± 18	0.2858	274 ± 8.3	273 ± 16	0.9085
Birthweight (g)	3398 ± 487	3445 ± 728	0.7976	3337 ± 475	3517 ± 676	0.2334

Data given as “mean ± standard deviation” or “count (%)”. Detailed demographics are available in Additional file 1

### DNA methylation analysis quality control

DNA methylation of first trimester human placentas was measured using the EPIC array. The fetal sex of processed placenta samples was verified experimentally using a principal components analysis starting with all sites on the EPIC array, including chromosomes X and Y which segregate samples by fetal sex (Fig. 1B, Additional file 2). However, sex chromosome DNA methylation cannot be accurately compared between female and male samples due to differences in copy numbers and phenomena such as X chromosome inactivation which involves wide spans of DNA and histone methylation across one copy of the female chromosome X, but not the other. Differentially methylated probes (DMPs) analysis was performed to identify sexually dimorphic sites using only autosomal chromosomes, without other sources of unreliability such as masked probes or probes at single nucleotide polymorphisms. Principal components analysis of the final sites shows that placental samples no longer cluster by fetal sex without the sex chromosomes, indicating that global placental DNA methylation is largely similar between sexes (Fig. 1B, Additional file 2). The Quantile–Quantile plot of P-values shows a lambda value of 1.1249 indicating low inflation (Fig. 1C).

### Differentially methylated probes (DMPs) affected by fetal sex

We identified 151 autosomal differentially methylated probes (DMPs) significantly altered between female and male placentas at FDR < 0.05 (here equal to P < 1 × 10^–5^), with a slight majority of 59% (89 DMPs) hypermethylated in females, and 62 hypermethylated in males (Additional file 3, Additional file 4). Among these 151 DMPs, there were 29 CpG sites that reached genome-wide significance (P < 9 × 10^–8^), with 10 hypermethylated in females and 19 hypermethylated in males (Table 2). The most significantly female hypermethylated probe was cg00167275, located in the 5′ untranslated region (5′UTR) and first exon of *glutamate dehydrogenase 1 (GLUD1)*, and before the transcription start site of double-strand break regulator *FAM35A* (also called *SHLD2*). The most significantly male-hypermethylated probe was cg17612569, located before the transcription start site of transcription factor subunit *GABPA* and at different locations of ATP synthase subunit *ATP5J* transcripts (5′UTR, first exon, and coding region). No CpH (CpA, CpC, or CpT) probes reached FDR < 0.05 significance.

**Table 2:** Differentially methylated probes (DMPs) in first trimester placenta that reach genome-wide significance (P < 9 × 10^–8^)

probeID	Chr:Start	UCSC gene (context)	Relation to UCSC CpG Island	LogFC and direction higher methylation		P-value	FDR
cg17612569	21: 27,107,221	GABPA (TSS200); ATP5J (5′UTR, 1stExon, Body)	Island	− 2.066	Male	4.58E−28	3.41E−22
cg13192180	17: 65,717,569	NOL11 (Body)	S_Shelf	− 0.902	Male	7.91E−18	2.94E−12
cg00167275	10: 88,854,588	FAM35A (TSS1500); GLUD1 (5′UTR, 1stExon)	Island	2.329	Female	5.36E−16	1.33E−10
cg15817705	1: 209,406,063		S_Shore	− 0.468	Male	5.33E−14	9.91E−09
cg09516963	12: 68,042,445	DYRK2 (TSS200)	Island	0.783	Female	4.41E−12	5.49E−07
cg09241427	11: 70,860,363	SHANK2 (5′UTR)		0.473	Female	4.43E−12	5.49E−07
cg08937153	8: 48,504,810	SPIDR (body)		− 0.602	Male	1.55E−11	1.65E−06
cg10661558	21: 15,443,159		Island	0.714	Female	7.50E−11	6.97E−06
cg00804338	13: 114,239,234	TFDP1 (5′UTR, Body)		1.629	Female	3.48E−10	2.87E−05
cg25452172	12: 58,162,751	METTL1 (3′UTR)	N_Shelf	− 0.330	Male	5.86E−10	4.06E−05
cg20374928	20: 42,087,910	SFRS6 (body)	S_Shore	0.467	Female	6.01E−10	4.06E−05
cg15604132	5: 150,285,117	ZNF300 (TSS1500)	S_Shore	− 1.277	Male	1.24E−09	7.70E−05
cg05994094	18: 31,020,806	C18orf34 (TSS200, TSS1500)	Island	1.698	Female	1.99E−09	0.000114
cg08580836	5: 150,284,448	ZNF300 (TSS200)	Island	− 1.782	Male	2.30E−09	0.000122
cg09829303	7: 134,419,774			0.380	Female	2.46E−09	0.000122
cg14974262	5: 150,284,594	ZNF300 (TSS200)	Island	−1.412	Male	4.76E−09	0.000217
cg04032578	5: 150,284,619	ZNF300 (TSS200)	Island	− 1.369	Male	4.96E−09	0.000217
cg12777992	5: 150,284,634	ZNF300 (TSS200)	Island	− 1.605	Male	5.75E−09	0.000237
cg21228005	5: 150,284,796	ZNF300 (TSS 1500)	S_Shore	− 2.192	Male	6.39E−09	0.00025
cg18237551	5: 150,284,600	ZNF300 (TSS 1500)	Island	− 1.469	Male	7.74E−09	0.000288
cg17561788	22: 20,114,683	RANBP1 (3′UTR)	N_Shelf	− 0.507	Male	1.10E−08	0.000387
cg12385553	22: 17,256,079			− 0.669	Male	1.15E−08	0.000387
cg02343823	5: 150,284,419	ZNF300 (TSS200)	Island	− 1.341	Male	1.73E−08	0.000558
cg07628841	2: 27,851,430	GPN1 (TSS200, TSS1500); CCDC121 (5′UTR, 1st Exon)		0.748	Female	1.90E−08	0.000587
cg11291313	5: 150,284,530	ZNF300 (TSS200)	Island	− 1.270	Male	2.10E−08	0.000626
cg09971754	16: 89,557,657	ANKRD11 (TSS1500)	Island	− 0.864	Male	2.57E−08	0.000736
cg13150358	2: 51,808,957			0.615	Female	3.77E−08	0.001017
cg26486466	5: 150,284,616	ZNF300 (TSS200)	Island	− 1.179	Male	3.83E−08	0.001017
cg21161307	8: 86,981,879			− 1.213	Male	7.03E−08	0.001802

*3′UTR* 3′ untranslated region, *5′UTR* 5′ untranslated region, *Chr* chromosome, *FDR* false discovery rate, *logFC* log fold change of methylation, *Start* start position within chromosome (GRCh37), *TSS200* window of 200 bases before the transcription start site, *UCSC* University of California Santa Cruz Genome Browser annotation for the probe

**Fig. 2:**
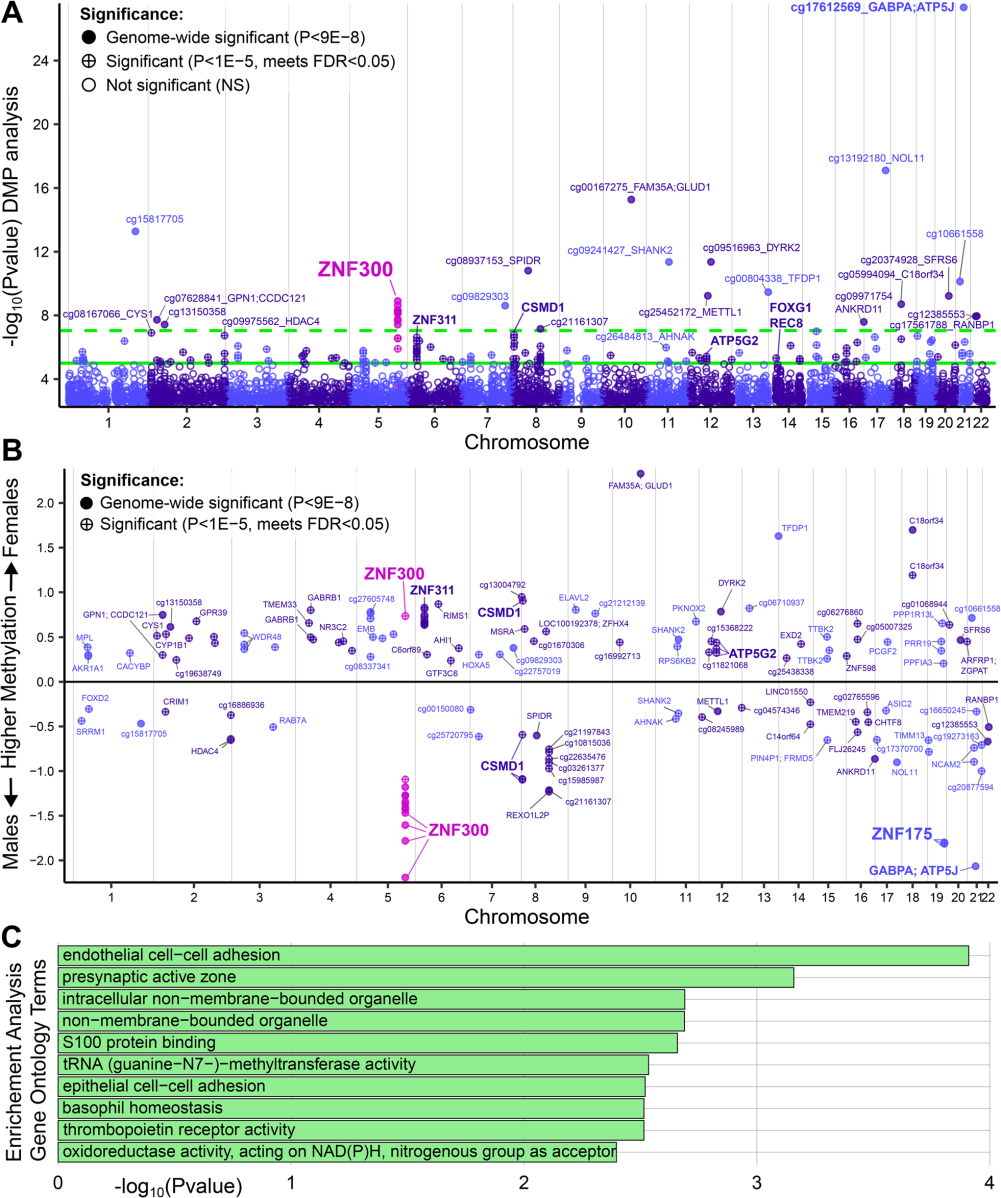
Manhattan plots of the top probes by P-values. Chromosomes alternate colors and magenta points highlight gene *ZNF300*. **A** The top 5000 probes by P-values separated by significance. Higher − log_10_(P) values means probes are more significantly sex different. Probes reach FDR < 0.05 (P < 1 × 10^–5^) significance above the solid green and genome-wide significance above the *dashed green line* at P = 9 × 10^–8^. **B** Only the significant probes (FDR < 0.05), plotted for direction of methylation: positive values indicate hypermethylation in female placentas, and *negative* values indicate hypermethylation in male placentas. **C** Top 10 enriched gene ontology terms for the DMPs, sorted by P-value

Manhattan plots were created to visualize site significance across the genome (Fig. 2A). Chromosome 5 showed a cluster of 13 probes mapped to the promoter region of *zinc finger protein 300* (*ZNF300)* gene, 200 bp or 1500 bp upstream of the *ZNF300* transcription start site (Additional file 4). Twelve sequential *ZNF300* probes were significantly hypermethylated in male placentas with strong trends, including 9 in a CpG island and 4 in a south shore (downstream of the CpG island), and only the final 13th probe (further downstream from the rest) showed female-hypermethylation. Additional clusters of methylation differences were evident across the genome, though not all reached genome-wide significance (Fig. 2B).

Gene ontology analysis was performed using the 151 DMPs, resulting in 248 enriched gene ontology terms at P < 0.05 (Additional file 5). The five most significant terms were “endothelial cell–cell adhesion” (P = 0.000123), “presynaptic active zone” (P = 0.000694), “intracellular non-membrane-bounded organelle” (P = 0.00204), “non-membrane-bounded organelle” (P = 0.002047), and “S100 protein binding” (P = 0.002195) (Fig. 2C).

### Differentially methylated regions

A detailed analysis of differentially methylated regions (DMRs) was performed to confirm findings on the Manhattan plot. We identified eleven DMRs on autosomal chromosomes, eight regions hypermethylated in female placentas and three regions hypermethylated in male placentas (Table 3). The most significant DMR was composed of 14 probes in *ZNF300*, hypermethylated in males. The longest DMR between female and male placentas was male-hypermethylated, located in genes *GABPA/ATP5J*, and composed of 23 probes across 1543 base pairs. The most significant female-hypermethylated DMR was located in *ZNF311* with 16 probes. Another zinc finger domain-containing transcription factor, *ZNF175*, appeared to be among the most male-hypermethylated genes (Fig. 2B) and was confirmed as a significant male-hypermethylated DMR. Chromosome 14 had three DMR, all female-hypermethylated, including two forkhead box transcription factor genes (*FOXG1* and *FOXA1*). Chromosome 6 had two DMRs (*ZNF311* and *C6orf47/C6orf47-AS1*), also both female-hypermethylated. Other DMRs were located on separate chromosomes.

**Table 3:** Differentially methylated regions (DMRs) between female and male placentas at first trimester

Genes	Chr	Start	End	Width	Mean differential/coefficient	Direction higher methylation	Number of probes	Min smoothed FDR
*ZNF300*	5	150,284,416	150,285,753	1338	− 0.180367513	Males	14	1.51E−101
*GABPA, ATP5J*	21	27,106,440	27,107,982	1543	− 0.010012545	Males	23	1.95E−97
*ZNF311*	6	28,972,876	28,974,115	1240	0.068131039	Females	16	1.44E−59
*CCDC178*	18	31,020,209	31,021,065	857	0.077019907	Females	10	9.97E−39
*ATP5G2*	12	54,070,357	54,071,264	908	0.05002285	Females	15	1.08E−34
*FOXG1*	14	29,235,904	29,236,535	632	0.071279022	Females	13	4.22E−34
*ZNF175*	19	52,073,990	52,075,189	1200	− 0.160994092	Males	10	3.35E−33
*SGCE*	7	94,284,258	94,284,919	662	0.040896097	Females	23	1.41E−31
*C6orf47-AS1, C6orf47*	6	31,628,050	31,628,440	391	0.047168157	Females	13	3.40E−25
*REC8*	14	24,640,895	24,641,201	307	0.062290522	Females	9	4.98E−25
*FOXA1*	14	38,064,490	38,064,532	43	0.02854228	Females	4	3.48E−24

*Chr* chromosome, *Start/end* chromosome positions in the hg19 (GRCh37) human genome context, *Min smoothed* *FDR* Minimum false discovery rate of the smoothed estimate

### Comparison to RNA-seq sex differences

**Fig. 3:**
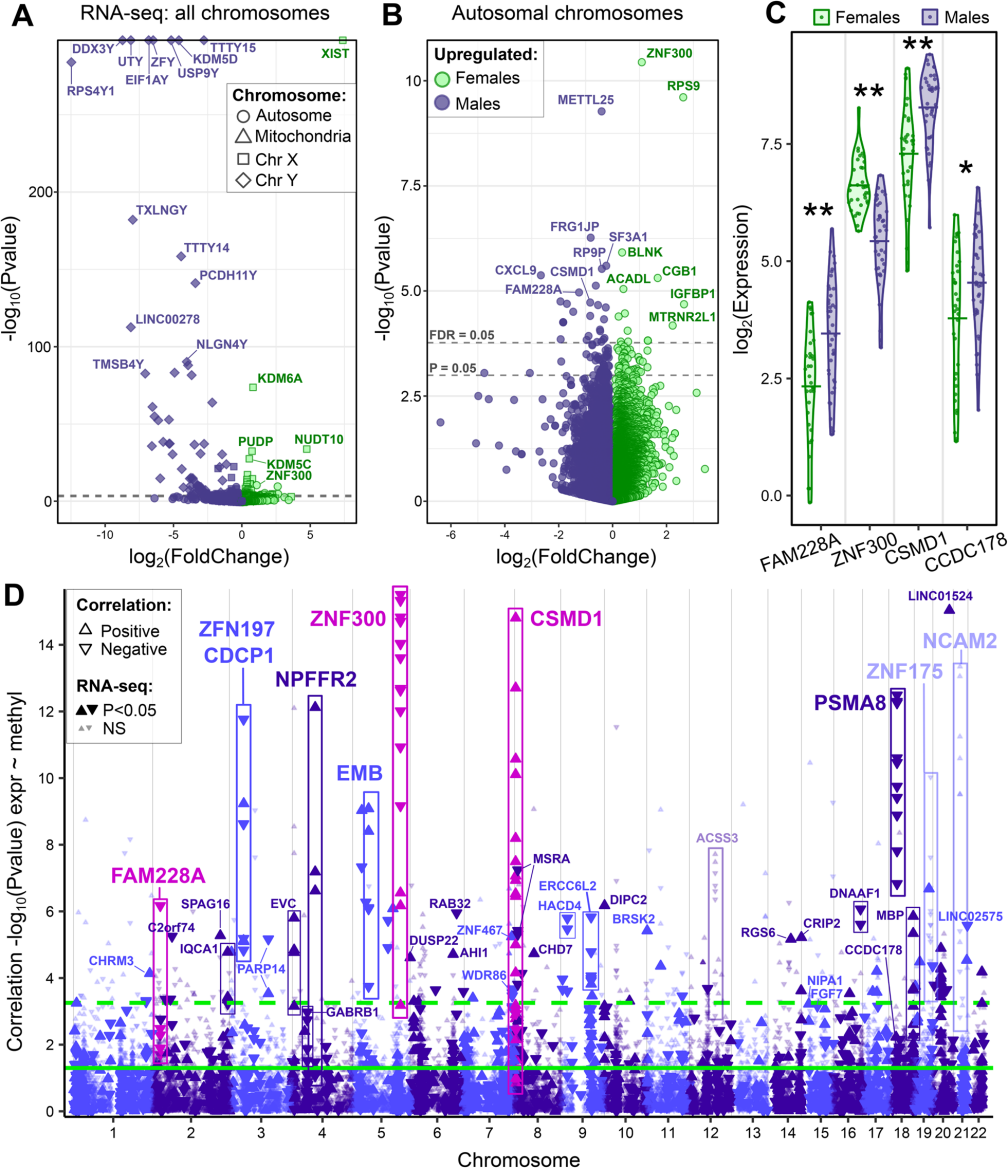
RNA-seq sex differences and correlation to DNA methylation. **A**, **B** Volcano plots for RNA-seq sex differences in placenta, with dashed lines at P = 0.05 and FDR = 0.05 (these lines overlap in A due to the y-axis range). **C** Violin plots comparing female and male gene expression: log2(baseMeans) adjusted for batch. Horizontal lines indicate the mean. Sex differences: *P < 0.05, **FDR < 0.05. **D** Manhattan plot of correlation results with the overlapping cohort (n = 51), comparing gene expression (“expr”) to DNA methylation (“methyl”). Each point is a probe. Probes located in the same gene are grouped with rectangles. Non-significant (“NS”, RNA-seq P ≥ 0.05) genes are semi-transparent. Magenta color highlights three genes with correlation FDR < 0.05 and RNA-seq FDR < 0.05 (*FAM228A*, *ZNF300* and *CSMD1*)

We previously found *ZNF300* gene expression significantly upregulated in female placenta, compared to male, in bulk total RNA-seq of n = 39 [9]. We combined additional bulk total RNA-seq data to perform a new sex differences analysis on n = 74 first trimester human placenta samples. Differential expression analysis identified 152 differentially expressed genes (DEGs) between females and males at FDR < 0.05, with 48 female-upregulated and 104 male-upregulated (Additional file 6). The most female-upregulated gene was *XIST* (167-fold higher in females, FDR = 0), an X-linked gene involved in X-chromosome inactivation. The most male-upregulated genes were Y-linked protein coding genes *RPS4Y1*, *DDX3Y*, *UTY*, *EIF1AY*, *ZFY*, *USP9Y*, *KDM5D*, and non-coding *TTTY15* (Fig. 3A)*.* By fold change, the most sexually dimorphic autosomal genes were *chemokine ligand 9* (*CXCL9*, 6.3-fold higher in males, FDR = 0.0021) and *insulin-like growth factor binding protein 1* (*IGFBP1*, 6.27-fold higher in females, FDR = 0.0090) (Fig. 3B). By significance, the most sexually dimorphic gene was *ZNF300* (2.12-fold higher in females, FDR = 2.98 × 10^–8^).

DMPs at FDR < 0.05 were matched to the RNA-seq results to identify overlapping genes of interest (Additional file 7). Of the 151 DMPs, 42 were located in genes that showed at least nominally significant sex differences in RNA-seq (P < 0.05), and 17 DMPs were located in two genes significant after adjustment for multiple comparisons (FDR < 0.05 in the RNA-seq): *ZNF300* which was consistently male-hypermethylated, and tumor suppressor gene *CUB and Sushi multiple domains 1 (CSMD1)* which had gene expression 1.78-fold higher in males but inconsistent CpG hypermethylation directions in the gene body (none in the promoter region). Probe *cg05994094* was genome-wide significant and hypermethylated in females and its corresponding gene, *coiled-coil domain-containing protein 178 (CCDC178),* had 1.46-fold higher gene expression in males at P < 0.05 (Fig. 3C), consistent with expectations. Three genes from the DMR region analysis were associated with sexually dimorphic gene expression: *ZNF300*, *ZNF311*, and *CCDC178.*

To directly access which DNA methylation sex differences affect gene expression, we integrated the datasets. DNA methylation probes with sex differences at P < 0.01 (12,560 probes) were matched to overlapping or nearby genes, resulting in 35,862 gene-probe matches for a correlation analysis using the 51 overlapping placentas (Additional file 8). Of these, 3083 (5.7%) and 396 (0.72%) gene-probe matches reached correlation significances of P < 0.05 or FDR < 0.05, respectively, indicating that methylation sex differences alter expression of a small subset of placental genes. When limited to sexually dimorphic genes identified earlier in the full cohort RNA-seq, 152 gene-probe matches (146 unique probes) show significant methylation correlation to gene expression in first trimester placenta (P < 0.01 DNA methylation, P < 0.05 RNA-seq, and FDR < 0.05 correlation). Of these, three genes met FDR < 0.05 in the RNA-seq: *FAM228A*, *ZNF300*, and *CSMD1* (Fig. 3C), indicating strong sex differences in the placental transcriptome*. FAM228A* showed a significant inverse (negative) correlation, indicating that female-hypermethylation causes male-upregulated expression (Fig. 3D). *ZNF300* showed an inverse correlation as well. *CSMD1* showed a positive correlation for the most significant 12 probes (correlating at FDR < 0.05), suggesting that hypermethylation at these sites increases gene expression. The correlation analysis also highlighted *proteasome subunit alpha type 8* (*PSMA8*) which showed a significant inverse correlation in eleven FDR < 0.05 probes. *ZNF175*, a sexually dimorphic DMR, showed strong inverse correlation between placental DNA methylation and gene expression (FDR < 0.05), though the gene was not significantly sex different in this RNA-seq.

### Methylation sex differences across studies

**Fig. 4:**
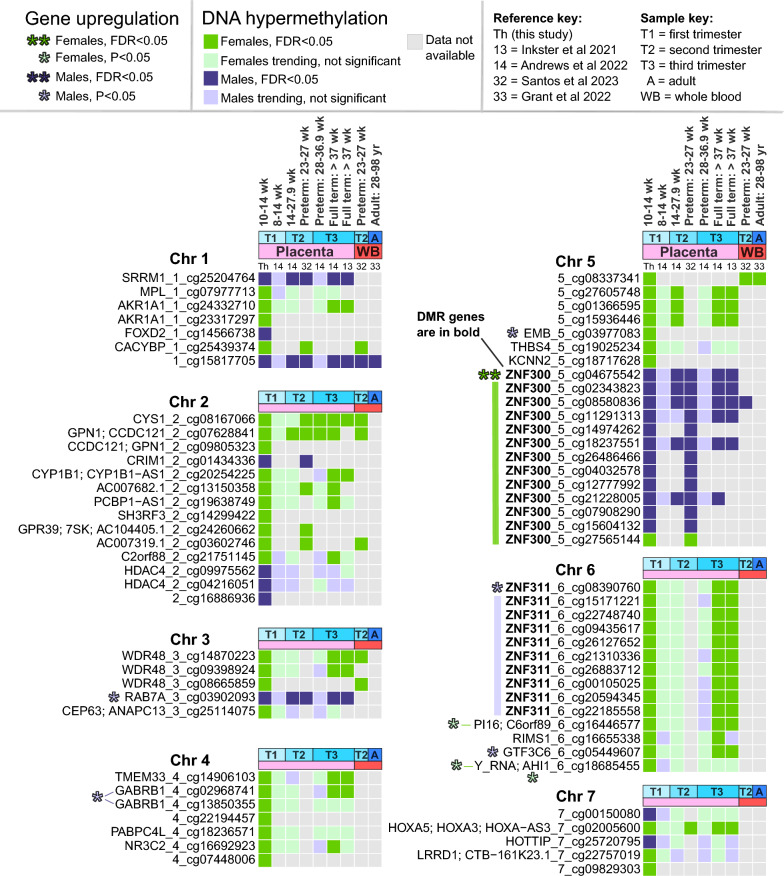
Comparison with other sex differences studies, chromosomes 1–7. DMPs FDR < 0.05 in this study are compared to DMPs in Andrews et al., Santos et al., Inkster et al., and Grant [13, 14, 32, 33]. DMPs are sorted by chromosome position and labeled “genes_chromosome_probeID”. RNA-seq sex differences in this study are highlighted for significant (FDR < 0.05) and nominally significant (P < 0.05) genes using asterisks, with color bars for genes with > 2 DMPs

**Fig. 5:**
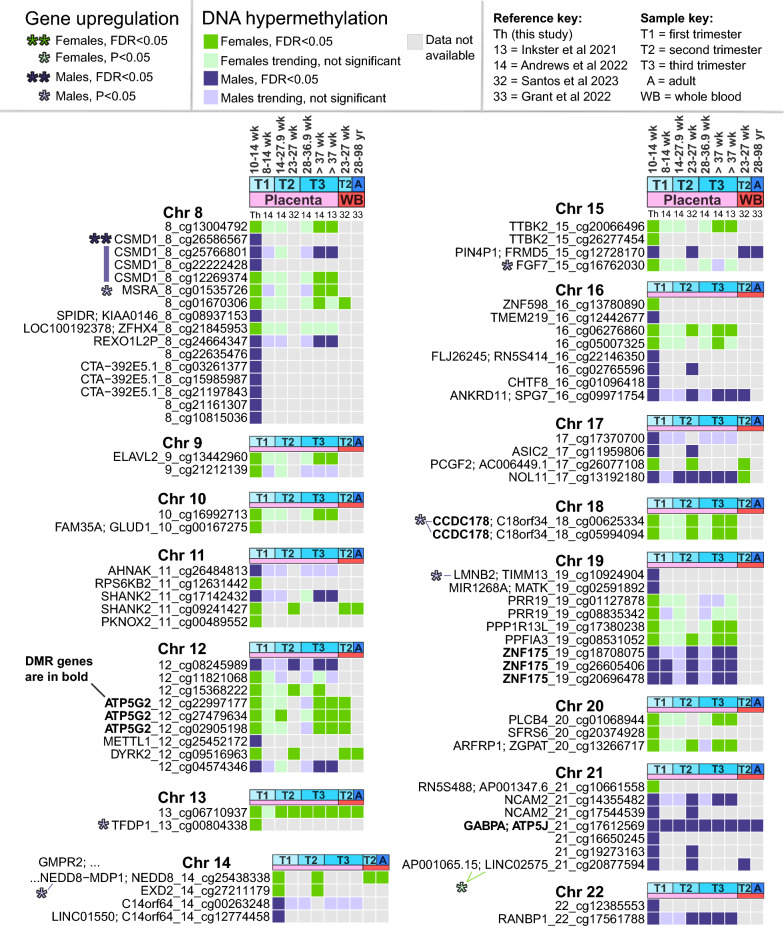
Comparison with other sex differences studies, chromosomes 8–22. Continuation of Fig. 4, with similar labeling

The 151 DMPs were cross-referenced across different studies to identify which are uniquely sex different in first trimester placenta, which show placental sex differences later in gestation, and which also show sex differences in the whole blood of neonates or adults (Figs. 4, 5). All studies were compared at significance threshold FDR < 0.05, and non-significant sites were examined for directional trends.

Our DMPs showed good consistency with Andrews et al. [14] sex differences analysis of human placenta at different gestational ages, including an independent cohort of 22 first trimester placentas at 8–14 weeks. We found novel results for 60 DMPs (40%) due to the higher number of methylation sites in the EPIC array, compared to the older 450 k array. Of 91 (60%) DMPs remaining, methylation directions were consistent across gestation: 84, 83, 66, and 86 DMPs matched in the first, second, third trimester (preterm), and full term placenta cohorts, respectively. At FDR < 0.05, we identified 3, 16, 6, and 71 DMPs with shared significance, respectively. The 3 DMPs significant in both first trimester cohorts were cg26605406 (*ZNF175*), cg20696478 (*ZNF175*), cg17612569 (*GABPA/ATP5J*), all male-hypermethylated genes significant in our region analysis (Table 2). We identified more significant matches with Andrews’ second trimester and full term cohorts, possibly because our cohort is at late first trimester (10–14 weeks). Inkster et al.’s [13] full term analysis includes some overlap with Andrews’ full term cohort and showed similar results, with 79 DMPs in the same direction (61 DMPs significant) and 66 DMPs with no available data. We also examined a sex-stratified comparison of second versus third trimester placenta [31], but no DMPs overlapped.

We also compared our 151 DMPs to two EPIC array studies of preterm placenta and whole blood. In Santos et al.’s [32] sex differences analysis of the Extremely Low Gestational Age Newborn (ELGAN) cohort (23–27 weeks, n = 1171), we found FDR < 0.05 matches for 51 DMPs in preterm placenta (34%) and 22 DMPs in neonatal peripheral blood (15%). In Grant et al.’s [33] adult blood analysis, only 8 DMPs (5%) matched, the lowest percent of all compared. One CpG methylation site was significantly male-hypermethylated at FDR < 0.05 in all studies including adult blood: cg17612569 (*GABPA/ATP5J*, chr21) (Fig. 5). Another near-unanimous methylation site was female-hypermethylated cg06710937 (chr13) near genes *RFESDP1*, *LINC00621*, *BASP1P1*, and *IPMKP1*, none of which were sex different in our placental RNA-seq.

Placental female-hypermethylation resulted in higher gene expression for males at several genes including: GABA-receptor subunit *GABRB1* (chr4), transmembrane glycoprotein *embigin (EMB*, chr5*)*, transcription factors *ZNF311* (chr6) and *GTF3C6* (chr6), *methionine sulfoxide reductase A (MSRA*, chr8), the cell cycle regulating transcription factor *TFDP1* (chr13), read-through product *NEDD8-MDP1* (chr14), *fibroblast growth factor 7* (*FGF7*, chr15), *CCDC178* (chr18). Of these transcriptome-altering DMPs, *MSRA* female-hypermethylation is uniquely significantly at first trimester placenta. *TFDP1* and *EMB* methylation may also be unique to first trimester placenta, but information is lacking across gestation. *CSMD1* DMPs (chr8) had inconsistent methylation directions, mostly male-hypermethylated except for female-hypermethylated cg12269374 located in the gene body. Two DMPs showed female-hypermethylation and female-upregulated gene expression: cg16446577 at peptidase inhibitor *PI16* (chr6) which was consistently female-hypermethylated across gestation, and cg18685455 (chr6) which corresponds to *AHI1* (involved in vesicle trafficking) as well as one of several regions encoding *Y_RNA* and is thus difficult to interpret.

Placental male-hypermethylation rarely resulted in higher gene expression for female placentas, only seen for our most significant result *ZNF300* (chr5), and for long non-coding gene *AP001065.15* also called *LINC02575* (chr21). *ZNF300* was consistently male-hypermethylated across gestation in placenta, but mostly not blood except for cg08580836 which was significant in neonatal blood [32]. *AP001065.15 (cg20877594)* was also male-hypermethylated in preterm placenta and matching neonatal blood [32]. Three other genes were male-hypermethylated in placenta but showed nominally higher gene expression in males: RAS oncogene family member *RAB7A* (chr3), *CSMD1* (chr8) discussed above, and nuclear *lamin B2* (*LMNB2*, chr19). *RAB7A* was male-hypermethylated across gestation in placenta, whereas *LMNB2* may be unique to first trimester placenta.

## Discussion

Global DNA methylation sex differences with associated gene expression differences are small in the first trimester placenta, however we identified 151 differentially methylated probes (DMPs) and 11 differentially methylated regions (DMRs) in first trimester placenta. Since we conducted DNA methylation and RNA-seq analysis on the same cohort, we were able to determine if changes in DNA methylation coincided with changes in gene expression in the same samples. Of interest, most significant DMPs and DMRs do not cause gene expression changes in the genes where they are localized, with only 42 DMPs and 3 DMRs associated with sexually dimorphic expression. This suggests that upstream mechanisms other than DNA methylation may play a role in sexually dimorphic gene regulation in the first trimester placenta during placentation.

Three of the 11 DMRs were associated with zinc finger protein genes (*ZNF300*, *ZNF311*, *ZNF175*), encoding DNA-binding proteins that often function as transcription factors. Our most significant result was male-hypermethylated *ZNF300*, found significantly sexually dimorphic in multiple placental DNA methylation and RNA-seq studies across gestation, including our previous RNA-seq study as well as other independent studies [9, 10, 13, 14, 34]. Placenta sex differences studies where it was not significant either could not evaluate (P-value = no value) or did not include it in the final analysis due to low gene expression in placenta [10, 11, 28]. *ZNF300*, though poorly expressed, may contribute to sexually dimorphic pregnancy outcomes. Hypermethylation of the *ZNF300* gene in placenta is associated with intrauterine growth restriction in twin pregnancies at term [35], as well as preeclampsia at term [36]. *ZNF300* is also involved in cellular differentiation, with knockdown of *ZNF300* leading to increased proliferation but decreased differentiation of megakaryocytes and erythrocytes from a progenitor cell line [37]. *ZNF300* therefore could be involved in adequate differentiation of placenta cells leading to proper establishment of the maternal-placental unit, though more research is needed. *ZNF175* was also significantly male-hypermethylated at first trimester here and a previous smaller study, but not significant in the second trimester and results were mixed at term [13, 14]. Although gene expression trended weakly higher in females in our first trimester (this study) and Olney et al.’s third trimester total RNA-seq [31], *ZNF175* only reached P < 0.1 and was not significant after adjustment for multiple comparisons in either trimester’s total RNA-seq. However, in our larger independent cohort analysis of messenger RNA (mRNA)-seq placental sex differences, we do find *ZNF175* significantly 1.4-fold higher in female first trimester (FDR = 5.35E−6), but not significant in third trimester (P > 0.2) [34]. This suggests that *ZNF175* sex differences in placental gene expression exist in early pregnancy but are more subtle, possibly only seen in polyadenylated transcripts or larger datasets. Differential methylation of *ZNF175* may play a role in sexually dimorphic placental disease states. *ZNF175* is hypomethylated and overexpressed in placenta of patients with preeclampsia, compared to controls [38–40]. Fetal sex appears to have a gestational age-dependent effect on preeclampsia risk, with female fetal sex more associated with preterm preeclampsia [6, 41], and otherwise conflicting results depending on study population [6, 42–44]. *ZNF311* was the most significantly female-hypermethylated DMR and showed significantly higher gene expression in males at first trimester, but loses sexual dimorphism by term [13, 14]. There is limited information regarding the role of *ZNF311*. It is an oncogene associated with tumor infiltrating immune cells in gliomas [45], and thus may play a role in the immune milieu at the maternal–fetal interface.

Genes with differentially methylated CpG sites and corresponding differential expression, unique to our dataset in the first trimester placenta, include *EMB*, *LMNB2*, *NEDD8-MDP1*, and *TFDP1*. The genes *EMB* and *TFDP1* contained female-hypermethylated sites and showed male upregulated gene expression in first trimester placenta. Both *EMB*, which encodes the Embigin transmembrane glycoprotein, and *TFDP1* which encodes a member of a family of transcription factors which control cell cycle regulators, are involved in cell growth which may play a role in sexual dimorphic outcomes in fetal growth [46]. Additionally, knockout of *TFDP1* with CRISPR/Cas9 reduces the invasion phenotype of HTR8 cells, a model of placental extravillous trophoblasts [47], and thus *TFDP1* alterations may directly affect placentation. *LMNB2* encodes lamin B2, a nuclear-localized protein involved in cell proliferation and apoptosis [48]. We found *LAMNB2* gene to be male-hypermethylated with higher gene expression in females. Abnormal expression of LMNB2 has been identified in multiple types of cancers, likely due to the impact on cellular proliferation and DNA damage repair [48]. Differential gene expression among the sexes may lead to differences in nuclear membrane stability, particularly in syncytiotrophoblasts, and cellular proliferation leading to alterations in placental function.

In first trimester, *CSMD1* gene was male-hypermethylated and showed a positive correlation with gene expression. *CSMD1* coverage in other placental array studies is poor and inconsistent. However, consistent with our results in first trimester, Gong et al.  who used term placenta and bisulfite sequencing found male-hypermethylation of *CSMD1*, then confirmed higher gene expression in males [49]. Furthermore, they found placenta-specific *CSMD1* gene regulation, with DNA methylation only sexually dimorphic in placenta but not eight other somatic tissues, and a placenta-specific transcript that starts downstream of the canonical *CSMD1* transcription start site. The DMPs we identified in first trimester placenta are still within the gene body and not the promoter, indicating an alternative gene regulation method than the typical gene suppression by promoter methylation. *CSMD1* gene expression has multiple tumor-suppressing functions through the SMAD pathway (promotes apoptosis, reduces cell proliferation and migration) [50], likely contributing to trophoblast cell regulation during placentation in first trimester. Notably, a polymorphism in *CSMD1* (rs2924725) affects COVID-19 hospitalization risk differently in females and males [51]. This suggests that the function of *CSMD1* is vulnerable to site-specific differences (either by DNA methylation or polymorphism) and that the downstream effect is further influenced by sex.

The most significant pathways of the DMPs included two cell–cell adhesion pathways. Cell–cell adhesion is an essential pathway for placentation which involves many categories of adhesion molecules (e.g. integrins) involved in within-placenta cell adhesion such as establishment of the syncytiotrophoblasts layer [52], as well as maternal–fetal interactions such as the development of the spiral arteries necessary for oxygen and nutrient transfer during pregnancy [53, 54]. Endothelial and epithelial cell–cell adhesion regulation specifically is essential for establishment of placental vascular networks, for example when trophoblasts (epithelial) invade uterine spiral uterine arteries (which begin with an endothelial lining) and restructure the cell environment [55, 56]. Defects or subtle alterations in cell–cell adhesion can contribute to the development of preeclampsia [54].

This placenta DNA methylation sex differences study has several strengths, including a larger single cohort and a primary focus on characterizing first trimester placenta. The study was designed to minimize confounding variables that affect methylation measurements. All placenta samples in this study were normal karyotype, collected within a short window of gestational age (10–14 weeks) in the same hospital, with DNA and RNA simultaneously co-isolated from the same tissue per subject, and DNA methylation measurements performed using the same array by the same facility for all samples. Similarly, RNA-seq was performed in the same facility for all samples. Previous placenta DNA methylation sex differences studies primarily focused on term placenta, with study designs and sample filtering most rigorous for term placenta [13, 14]. Due to challenges acquiring early pregnancy placenta, when first trimester samples were included in previous studies, they were analyzed from combined datasets of smaller studies performed on different methylation arrays (450k or EPIC), with wider allowances for gestational age (8–14 weeks) or no gestational age specified beyond trimester, and inconsistent tissue processing [14, 57, 58]. These allowances were necessary to combine enough first trimester samples for preliminary analysis, since first trimester samples are very limited, but this follow up study was necessary. This work provides improved datasets and analyses of placental sex differences at first trimester, specifically within the window of placentation which is a critical timepoint for pregnancy development, for both DNA methylation and total RNA-seq. Furthermore, all pregnancies resulted in live births.

We acknowledge study limitations such as a lack of third trimester samples, advanced parental ages, and limited racial diversity (most subjects were Caucasian). Placental DNA methylation sex differences are already well-characterized at later pregnancy and we benefited from prior work. Advanced parental ages are due to the source of first trimester placenta which was leftover tissue from prenatal diagnostic testing. Advanced maternal age does not significantly affect global DNA methylation in first trimester placenta, though individual regions are altered [59]. Parental age, race and ethnic diversity is limited by biobank availability, though we have shown here and in another mRNA-seq study that placenta samples did not separate by parental ages or race [60]. Fetal ethnicity (Hispanic versus non-Hispanic) may affect DNA methylation and future studies with larger cohorts are needed to explore this.

## Perspective and significance

This study is the most comprehensive analysis of fetal sex and its effect on human placental DNA methylation and gene expression. Prior studies show that sex differences affect pregnancy development and outcomes, but placenta studies at early pregnancy are limited and often subject to batch effects. We characterized DNA methylation using a single unified cohort and the most current and comprehensive human DNA methylation array available (the Infinium MethylationEPIC BeadChip array, with twice as many sites as the Infinium HumanMethylation450k BeadChip array). We performed differential gene expression analysis with an overlapping cohort and identified genes with DNA methylation differences and corresponding gene expression changes. We compared our results to other sex differences studies and found that placental sex differences across gestation were largely consistent in direction of hypermethylation, though not always in significance. Several genes were also significant in term placenta studies, showing that fetal sex alters placental epigenetics and gene expression early and that some changes are sustained until delivery. Comparisons with blood methylation studies found that placental sex differences best matched neonatal blood, but not adult blood, further indicating that some sex differences are age-dependent. We also identified genes with DNA methylation sex differences uniquely present in first trimester placenta, not seen later in gestation or in blood. These unique differences may be important for placentation and other processes of early pregnancy. Future work is needed to understand the specific functions of identified genes within the context of early pregnancy and placenta, as most of these genes have been studied in other organs in non-pregnant subjects.

### Supplementary Information

Additional file 1. Demographics analysis by fetal sex. Demographics for the placenta DNA methylation (Table S1, n = 56) and RNA-seq (Table S2, n = 74) cohorts.

Additional file 2. Principal Components Analyses. Plots for DNA methylation (all probes including those on sex chromosomes), DNA methylation (only autosomal probes), and RNA-sequencing (all chromosomes). Subjects were color-coded by (A, B) fetal sex, (C) gestational age at time of chorionic villus sampling, (D) maternal age, (E) paternal age, (F) maternal BMI, (G) gestational age at delivery, (H, I) fetal ethnicity, (J) fetal race. White points indicate unavailable demographics.

Additional file 3. Differentially methylated probe (DMP) analysis results for all 743,461 methylation sites analyzed.

Additional file 4. Genome context for DMPs with sex differences at P < 0.01 in human first trimester placenta, including the 151 DMPs significant at FDR < 0.05.

Additional file 5. Gene Ontology (GO) analysis using the 151 DMPs.

Additional file 6. Total RNA-seq sex differences in first trimester human placenta, adjusted for two sequencing runs. DESeq2 results annotated with Ensembl release 91. Sex and batch details are included for each subject.

Additional file 7. Gene expression matches for DMPs. The 151 DMPs with significant sex differences (FDR < 0.05) were matched to total RNA-seq sex differences results (any P value). ProbeIDs were matched to Ensembl Gene IDs.

Additional file 8. Correlation analysis comparing DNA methylation data for select probes (with sex differences at P < 0.01) and gene expression from RNA-seq (any Ensembl Gene IDs matching those probes).

## Data Availability

The dataset for DNA methylation will be made available after manuscript acceptance. Total RNA-seq data is available at The National Center for Biotechnology Information Gene Expression Omnibus (NCBI GEO) under accession GSE215421.
