# Supplementary material for: Sexually dimorphic DNA methylation and gene expression patterns in human first trimester placenta
Source: Biol Sex Differ. 2024 Aug 16;15:63. doi: 10.1186/s13293-024-00629-9 (PMC11328442; doi:10.1186/s13293-024-00629-9)
Supplement: Supplementary file 1 — Additional file 1. Demographics analysis by fetal sex. Demographics for the placenta DNA methylation (Table S1, n = 56) and RNA-seq (Table S2, n = 74) cohorts. [file 13293_2024_629_MOESM1_ESM.docx]

**Additional File 1**

**Supplemental Table S1.** Demographics for the placenta DNA methylation cohort (n=56 pregnancies), 25 females and 31 males.

| *DNA methylation cohort* | Females  (n=25) | Males  (n=31) | P-value | Stat test | Number of obs. |
| --- | --- | --- | --- | --- | --- |
| BASELINE DEMOGRAPHICS | | | |  |  |
| Maternal age (years) | 40 ± 1.9  (r: 37-43, 45) | 39 ± 2.2  (r: 34, 35-43) | 0.1003 | t-test | 56 |
| Paternal age (years) | 42 ± 5.8  (r: 25, 29, 36-50) | 41 ± 4.0  (r: 34-49) | 0.2862 | t-test | 56 |
| Maternal race |  |  | 0.497 | chi | 56 |
| Caucasian | 23 (92%) | 29 (94%) |  |  |  |
| Asian | 1 (4%) | 2 (6.5%) |  |  |  |
| African American | 1 (4%) | 0 |  |  |  |
| Biracial  (Caucasian/Asian) | 0 | 0 |  |  |  |
| Biracial (Other) | 0 | 0 |  |  |  |
| Maternal ethnicity  (non-Hispanic) | 22 (88%) | 29 (94%) | 0.469 | chi | 56 |
| Paternal race |  |  | 0.186 | chi | 54 |
| Caucasian | 19 (79%) | 29 (97%) |  |  |  |
| Asian | 2 (8.3%) | 1 (3.3%) |  |  |  |
| African American | 2 (8.3%) | 0 |  |  |  |
| Biracial  (Caucasian/Asian) | 0 | 0 |  |  |  |
| Biracial (Other) | 1 (4.2%) | 0 |  |  |  |
| Paternal ethnicity  (non-Hispanic) | 21 (84%) | 29 (94%) | 0.251 | chi | 56 |
| Fetal race |  |  | 0.398 | chi | 56 |
| Caucasian | 20 (80%) | 29 (95%) |  |  |  |
| Asian | 1 (4%) | 1 (3.2%) |  |  |  |
| African American | 1 (4%) | 0 |  |  |  |
| Biracial  (Caucasian/Asian) | 1 (4%) | 1 (3.2%) |  |  |  |
| Biracial (Other) | 2 (8%) | 0 |  |  |  |
| Multiracial | 0 | 0 |  |  |  |
| Fetal ethnicity  (non-Hispanic) | 18 (72%) | 27 (87%) | 0.157 | chi | 56 |
| Maternal BMI (kg/m^2^) | 23 ± 3.8 | 22 ± 2.8 | 0.3908 | t-test | 55 |
| Any Maternal Conditions | 8 (32%) | 5 (16%) | 0.162 | chi | 56 |
| Hypertension | 0 | 1 (3.2%) | 0.365 | chi | 56 |
| Diabetes | 0 | 0 |  | chi | 56 |
| Thyroid disease | 7 (28%) | 4 (13%) | 0.157 | chi | 56 |
| Other conditions | 1 (4%) | 0 | 0.261 | chi | 56 |
| PREGNANCY OUTCOMES | | | | |  |
| Gestational age at CVS (days) | 82 ± 7.3  (r: 71-95, 99) | 82 ± 5.5  (r: 73-89, 96) | 0.8851 | t-test | 56 |
| Crown rump length at CVS (mm) | 55 ± 12 | 55 ± 12 | 0.8906 | t-test | 53 |
| Gestational age at delivery (days) | 276 ± 7.5  (r: 262-291, 295) | 272 ± 18  (r: 207, 230, 261-301) | 0.2858 | t-test | 51 |
| Birthweight (grams) | 3398 ± 487 | 3445 ± 728 | 0.7976 | t-test | 47 |
| Mode of delivery |  |  | 0.627 | chi | 52 |
| Vaginal | 15 (65%) | 17 (59%) |  |  |  |
| Cesarean Section | 8 (35%) | 12 (41%) |  |  |  |
| Pregnancy complications | 2 (8%) | 5 (16%) | 0.361 | chi | 56 |
| Hypertension | 0 | 1 (3.2%) | 0.365 | chi | 56 |
| Gestational  Diabetes | 1 (4%) | 4 (13%) | 0.245 | chi | 56 |
| Coagulation disorders | 0 | 0 |  | chi | 56 |
| Placenta previa | 1 (4%) | 0 | 0.261 | chi | 56 |
| Placental abruption | 0 | 1 (3.2%) | 0.365 | chi | 56 |
| Placenta other | 0 | 0 |  | chi | 56 |

Data given as “count (%)” or “mean ± standard deviation” or “mean ± standard deviation (range: ## - ## with outliers noted outside the range)”. For example, in male gestational age at delivery, two births were preterm and the rest were full term (>37 weeks). The two preterm births were not excluded from the study because this is an outcome associated with the study variable (fetal sex affects risk of preterm birth). Some ranges were not given for privacy reasons, e.g. maternal BMI, crown rump length, and birthweight. CVS = chorionic villus sampling. Obs = observations. Stat test = statistical test used, either t-test or Chi-squared test.

**Supplemental Table S2.** Demographics for the placenta RNA-seq cohort (n=74 pregnancies), 34 females and 40 males.

| *RNA-seq cohort* | Females  (n=34) | Males  (n=40) | P-value | Stat test | Number of obs. |
| --- | --- | --- | --- | --- | --- |
| BASELINE DEMOGRAPHICS | | | |  |  |
| Maternal age (years) | 39 ± 2.0 (r: 36-42, 45) | 39 ± 2.4 (r: 34-43) | 0.4216 | t-test | 74 |
| Paternal age (years) | 41 ± 5.4  (r: 25, 29, 32-50) | 41 ± 4.6  (r: 29, 34-49) | 0.7726 | t-test | 74 |
| Maternal race |  |  | 0.462 | chi | 74 |
| Caucasian | 32 (94%) | 39 (98%) |  |  |  |
| Asian | 2 (5.9%) | 1 (2.5%) |  |  |  |
| African American | 0 | 0 |  |  |  |
| Biracial  (Caucasian/Asian) | 0 | 0 |  |  |  |
| Biracial (Other) | 0 | 0 |  |  |  |
| Maternal ethnicity  (non-Hispanic) | 29 (85%) | 37 (93%) | 0.320 | chi | 74 |
| Paternal race |  |  | 0.157 | chi | 72 |
| Caucasian | 30 (91%) | 39 (100%) |  |  |  |
| Asian | 2 (6.1%) |  |  |  |  |
| African American | 0 | 0 |  |  |  |
| Biracial  (Caucasian/Asian) | 1 (3.0%) |  |  |  |  |
| Biracial (Other) | 0 | 0 |  |  |  |
| Paternal ethnicity  (non-Hispanic) | 27 (79%) | 38 (95%) | 0.041 | chi | 74 |
| Fetal race |  |  | 0.055 | chi | 74 |
| Caucasian | 29 (85%) | 39 (98%) |  |  |  |
| Asian | 0 | 0 |  |  |  |
| African American | 0 | 0 |  |  |  |
| Biracial  (Caucasian/Asian) | 5 (15%) | 1 (2.5%) |  |  |  |
| Biracial (Other) | 0 | 0 |  |  |  |
| Multiracial | 0 | 0 |  |  |  |
| Fetal ethnicity  (non-Hispanic) | 24 (71%) | 35 (88%) | 0.071 | chi | 74 |
| Maternal BMI (kg/m^2^) | 22 ± 3.6 | 22 ± 2.8 | 0.5489 | t-test | 73 |
| Any Maternal Conditions | 10 (29%) | 8 (20%) | 0.347 | chi | 74 |
| Hypertension | 0 | 2 (5%) | 0.186 | chi | 74 |
| Diabetes | 0 | 0 |  | chi |  |
| Thyroid disease | 9 (26%) | 7 (18%) | 0.350 | chi | 74 |
| Other conditions | 1 (2.9%) | 0 | 0.275 | chi | 74 |
| PREGNANCY OUTCOMES | | | | |  |
| Gestational age at CVS (days) | 84 ± 6.4 (r: 71-95, 99) | 82 ± 5.9  (r: 73-92, 96) | 0.2350 | t-test | 74 |
| Crown rump length at CVS (mm) | 57 ± 11 | 55 ± 12 | 0.3508 | t-test | 71 |
| Gestational age at delivery (days) | 274 ± 8.3 (r: 255, 256,  262-291, 295) | 273 ± 16  (r: 207, 230, 260-301) | 0.9085 | t-test | 69 |
| Birthweight (grams) | 3337 ± 475 | 3517 ± 676 | 0.2334 | t-test | 62 |
| Mode of delivery |  |  | 0.660 | chi | 70 |
| Vaginal | 21 (66%) | 23 (61%) |  |  |  |
| Cesarean Section | 11 (34%) | 15 (39%) |  |  |  |
| Pregnancy complications | 4 (12%) | 6 (15%) | 0.685 | chi | 74 |
| Hypertension | 1 (2.9%) | 2 (5%) | 0.654 | chi | 74 |
| Gestational  Diabetes | 2 (5.9%) | 3 (7.5%) | 0.782 | chi | 74 |
| Coagulation disorders | 0 | 0 |  | chi | 74 |
| Placenta previa | 1 (2.9%) | 1 (2.5%) | 0.907 | chi | 74 |
| Placental abruption | 0 | 1 (2.5%) | 0.353 | chi | 74 |
| Placenta other | 0 | 0 |  | chi | 74 |

Data given as “count (%)” or “mean ± standard deviation” or “mean ± standard deviation (range: ## - ## with outliers noted outside the range)”. For example, in male gestational age at delivery, two births were preterm and the rest were full term (>37 weeks). The two preterm births were not excluded from the study because this is an outcome associated with the study variable (fetal sex affects risk of preterm birth). Some ranges were not given for privacy reasons, e.g. maternal BMI, crown rump length, and birthweight. CVS = chorionic villus sampling. Obs = observations. Stat test = statistical test used, either t-test or Chi-squared test.
