# Supplementary material for: Sexually dimorphic DNA methylation and gene expression patterns in human first trimester placenta
Source: Biol Sex Differ. 2024 Aug 16;15:63. doi: 10.1186/s13293-024-00629-9 (PMC11328442; doi:10.1186/s13293-024-00629-9)
Supplement: Supplementary file 2 — Additional file 2. Principal Components Analyses. Plots for DNA methylation (all probes including those on sex chromosomes), DNA methylation (only autosomal probes), and RNA-sequencing (all chromosomes). Subjects were color-coded by (A, B) fetal sex, (C) gestational age at time of chorionic villus sampling, (D) maternal age, (E) paternal age, (F) maternal BMI, (G) gestational age at delivery, (H, I) fetal ethnicity, (J) fetal race. White points indicate unavailable demographics. [file 13293_2024_629_MOESM2_ESM.pdf]

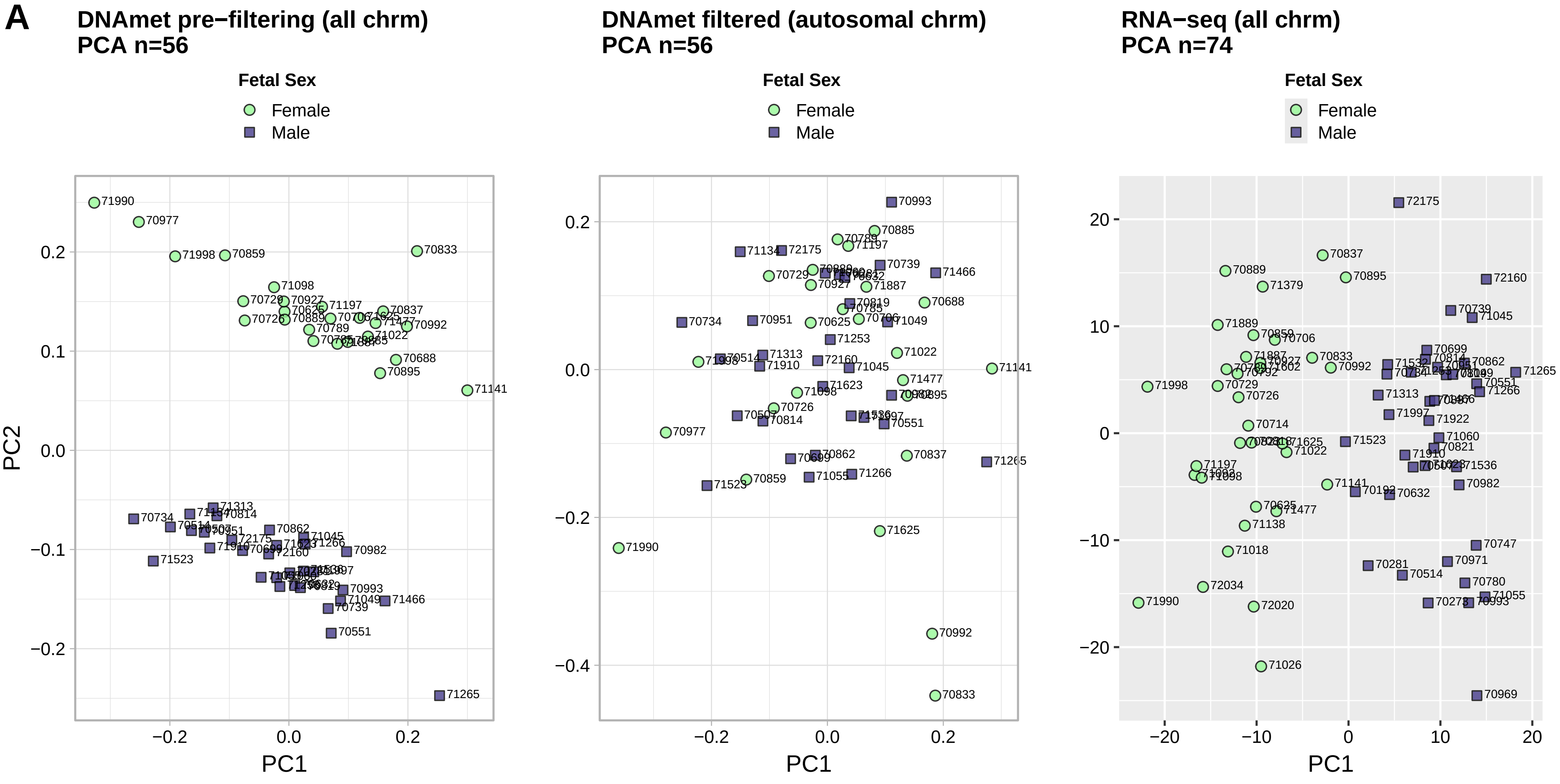
A
DNAmet pre−filtering (all chrm)
PCA n=56
Fetal Sex
Female
Male
PC2
PC1
DNAmet filtered (autosomal chrm)
PCA n=56
Fetal Sex
Female
Male
PC1
RNA−seq (all chrm)
PCA n=74
Fetal Sex
Female
Male
PC1

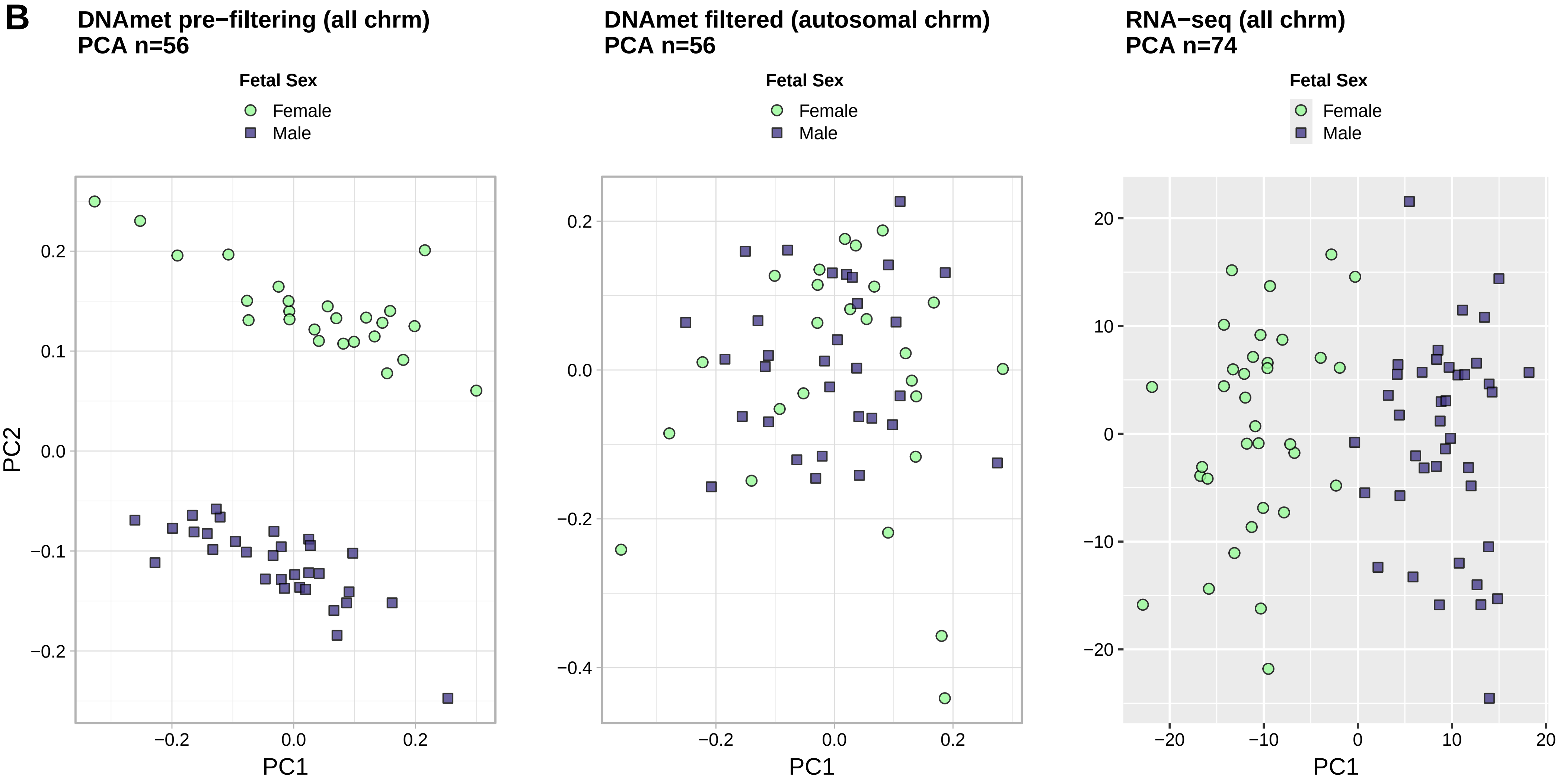
B
DNAmet pre−filtering (all chrm)
PCA n=56
Fetal Sex
Female
Male
PC2
PC1
DNAmet filtered (autosomal chrm)
PCA n=56
Fetal Sex
Female
Male
PC1
RNA−seq (all chrm)
PCA n=74
Fetal Sex
Female
Male
PC1

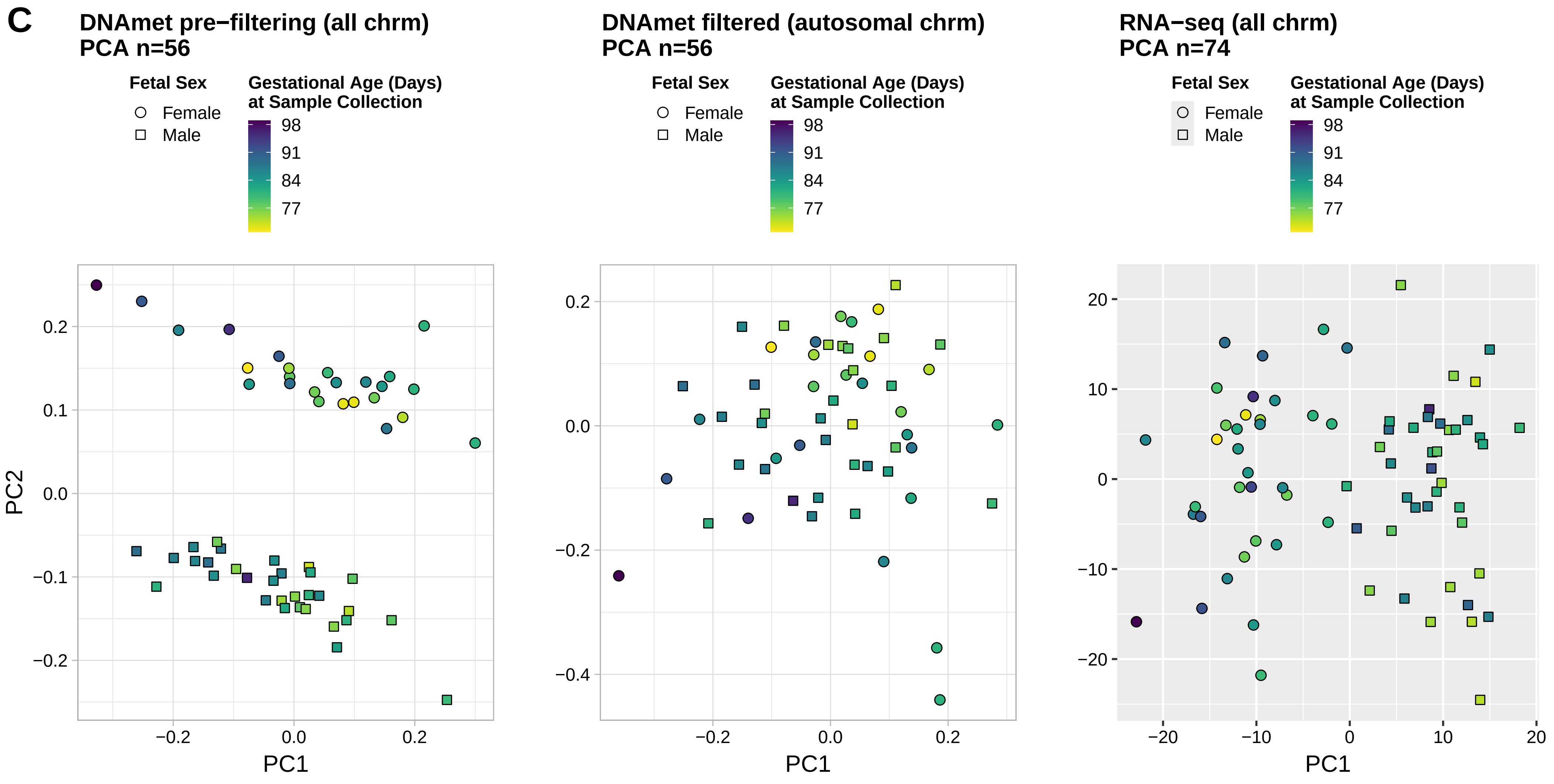
C
DNAmet pre−filtering (all chrm)
PCA n=56
Fetal Sex
Female
Male
Gestational Age (Days) at Sample Collection
98
91
84
77
PC2
PC1
DNAmet filtered (autosomal chrm)
PCA n=56
RNA−seq (all chrm)
PCA n=74

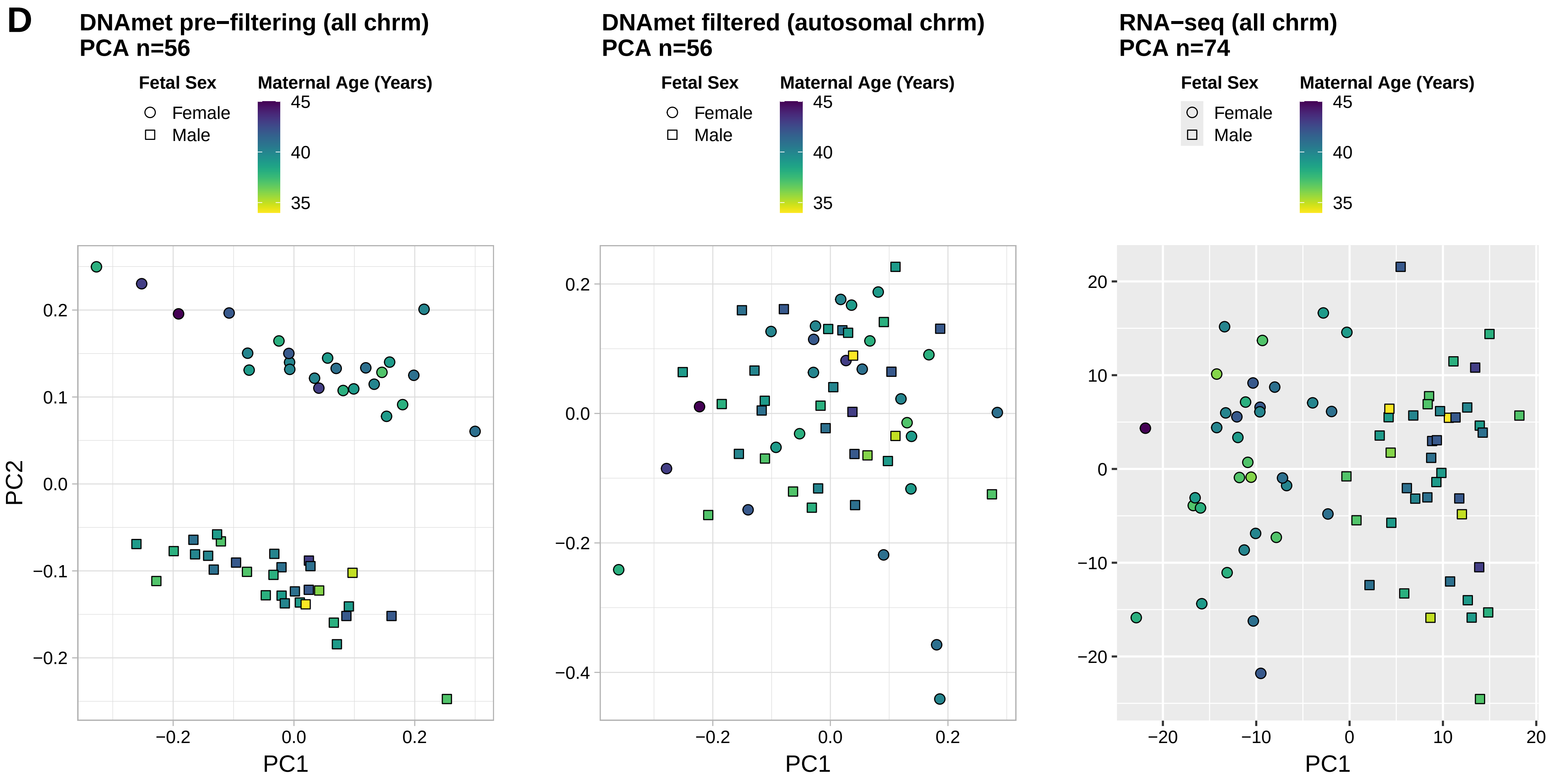
D
DNAmet pre−filtering (all chrm)
PCA n=56
DNAmet filtered (autosomal chrm)
PCA n=56
RNA−seq (all chrm)
PCA n=74
Fetal Sex
Female
Male
Maternal Age (Years)
45
40
35
PC2
PC1
0.2
0.1
0.0
−0.1
−0.2
−0.4
20
10
0
−10
−20

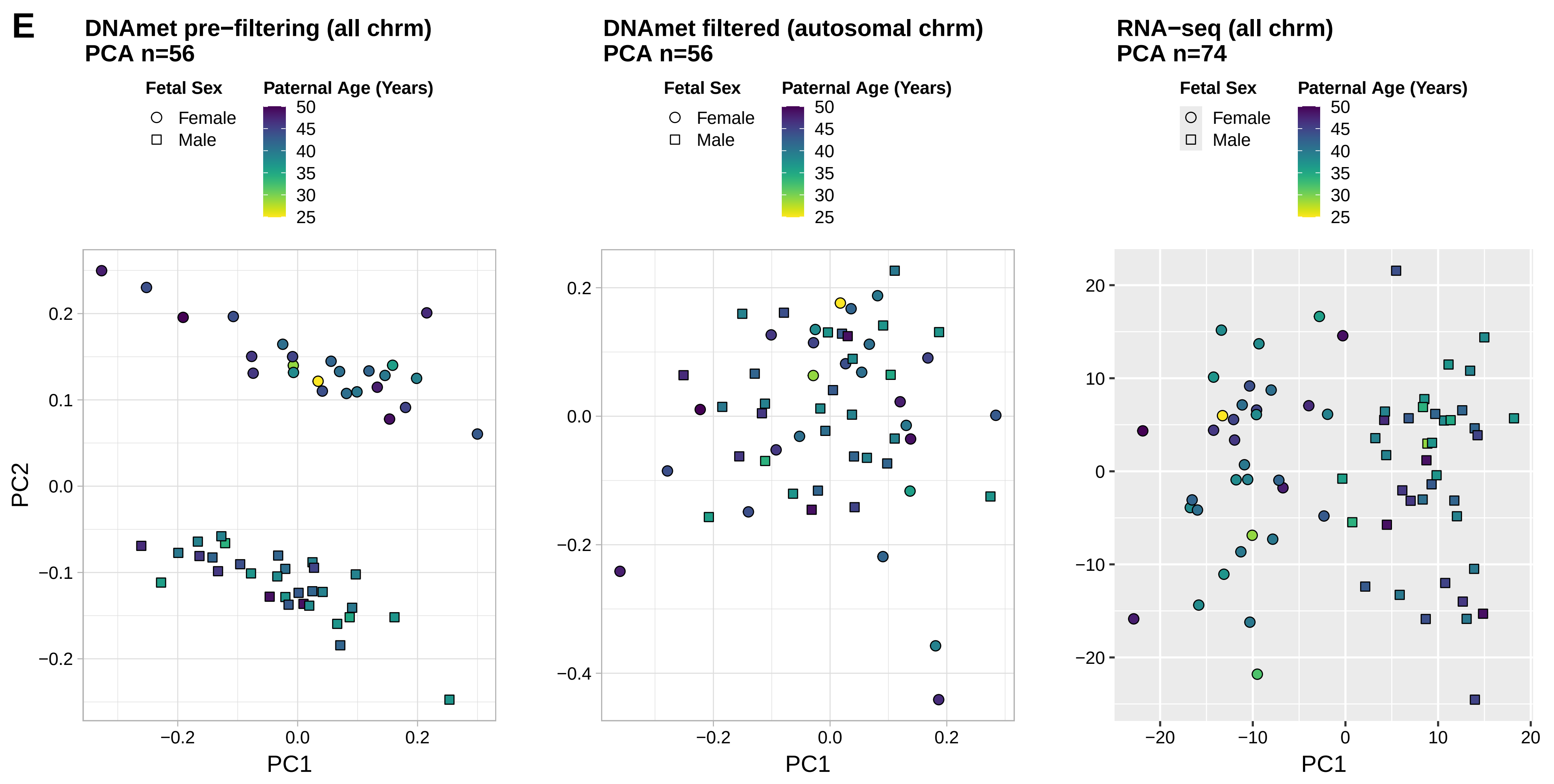
E
DNAmet pre−filtering (all chrm)
PCA n=56
DNAmet filtered (autosomal chrm)
PCA n=56
RNA−seq (all chrm)
PCA n=74
Fetal Sex
Female
Male
Paternal Age (Years)
50
45
40
35
30
25
PC1
PC2

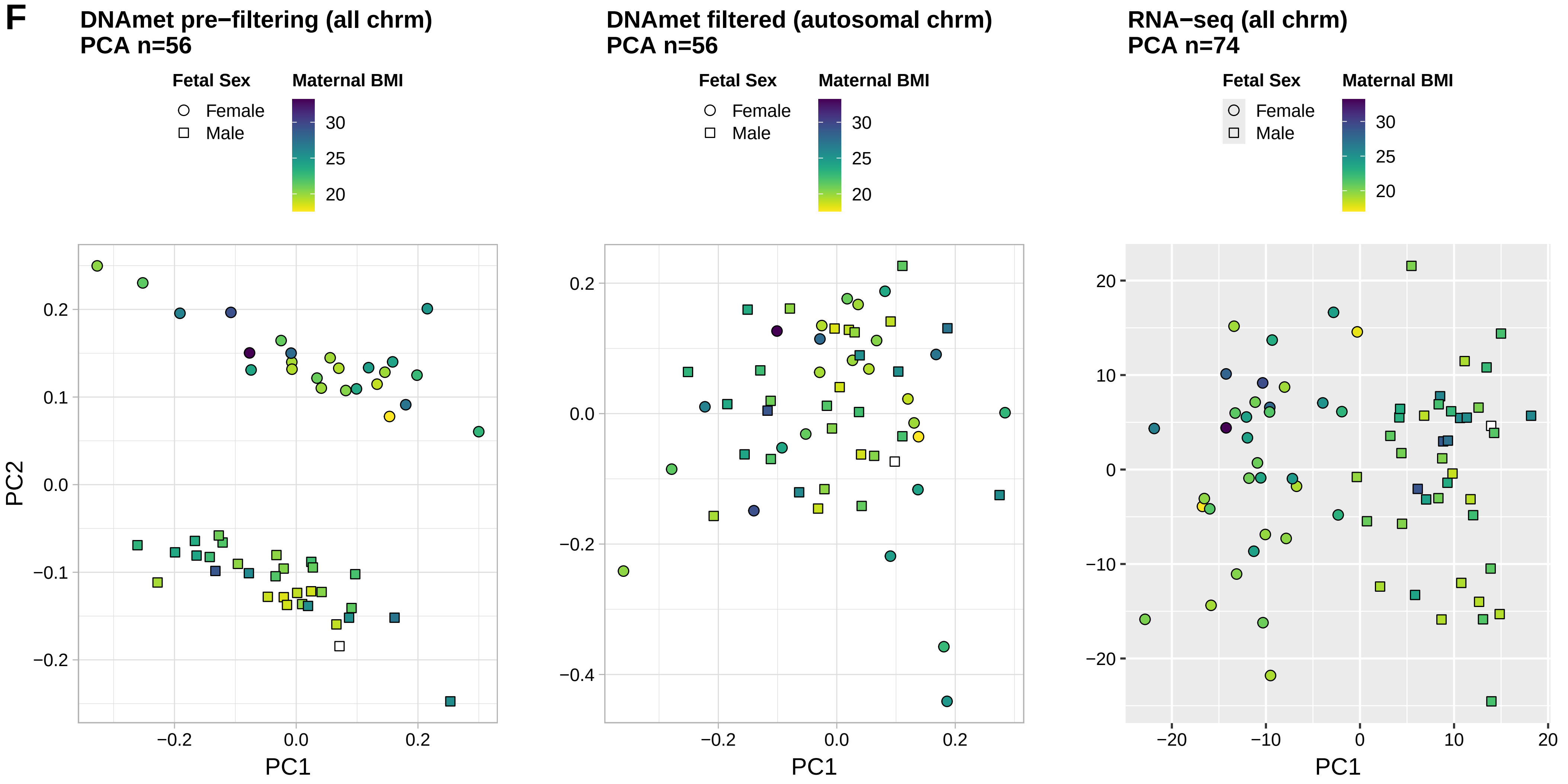

F
DNAmet pre−filtering (all chrm)
PCA n=56
Fetal Sex
Female
Male
Maternal BMI
30
25
20
PC2
PC1
DNAmet filtered (autosomal chrm)
PCA n=56
Fetal Sex
Female
Male
Maternal BMI
30
25
20
PC1
RNA−seq (all chrm)
PCA n=74
Fetal Sex
Female
Male
Maternal BMI
30
25
20
PC1

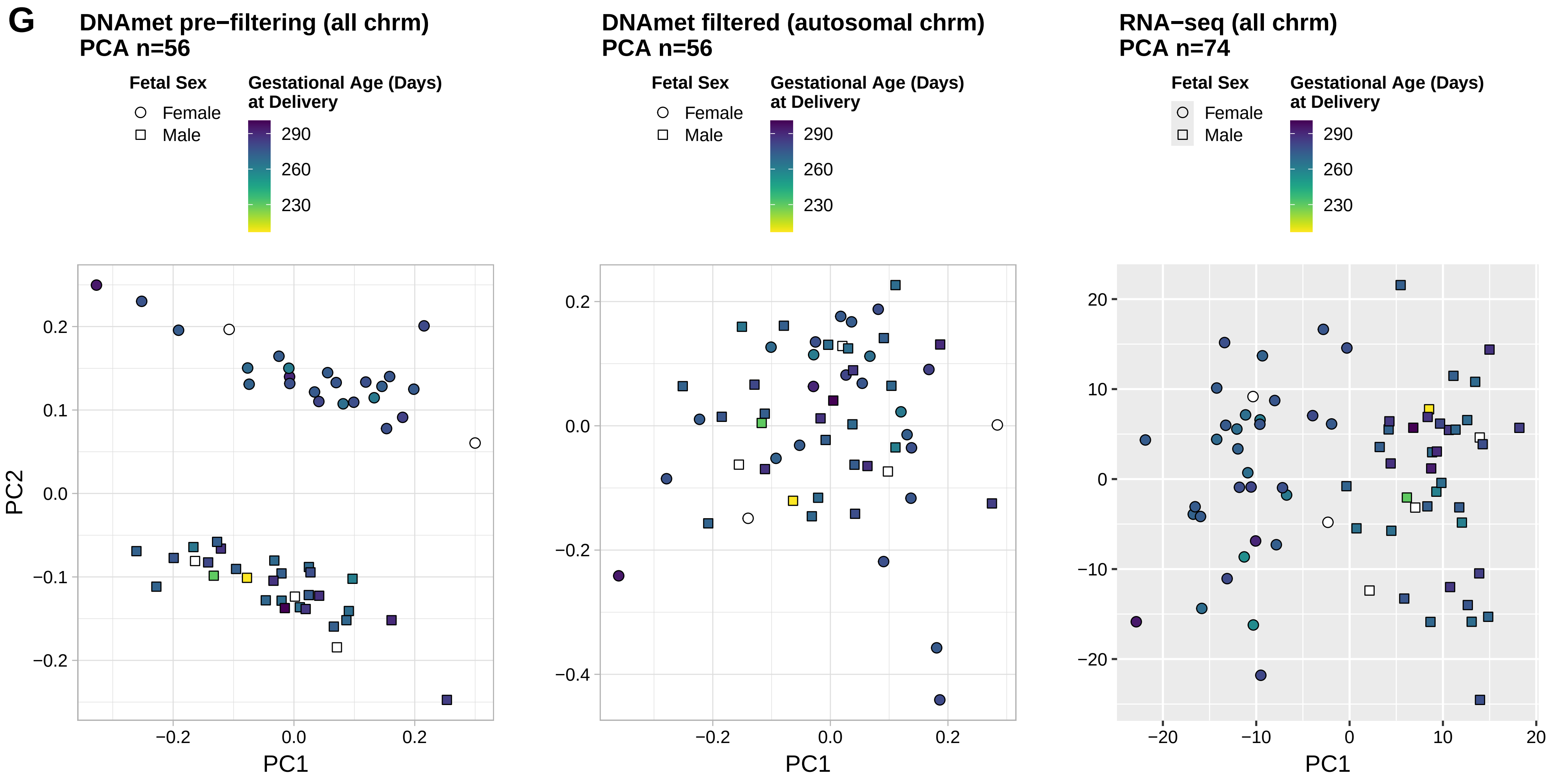
G
DNAmet pre−filtering (all chrm)
PCA n=56
DNAmet filtered (autosomal chrm)
PCA n=56
RNA−seq (all chrm)
PCA n=74
Fetal Sex
Female
Male
Gestational Age (Days) at Delivery
290
260
230
PC1
PC2

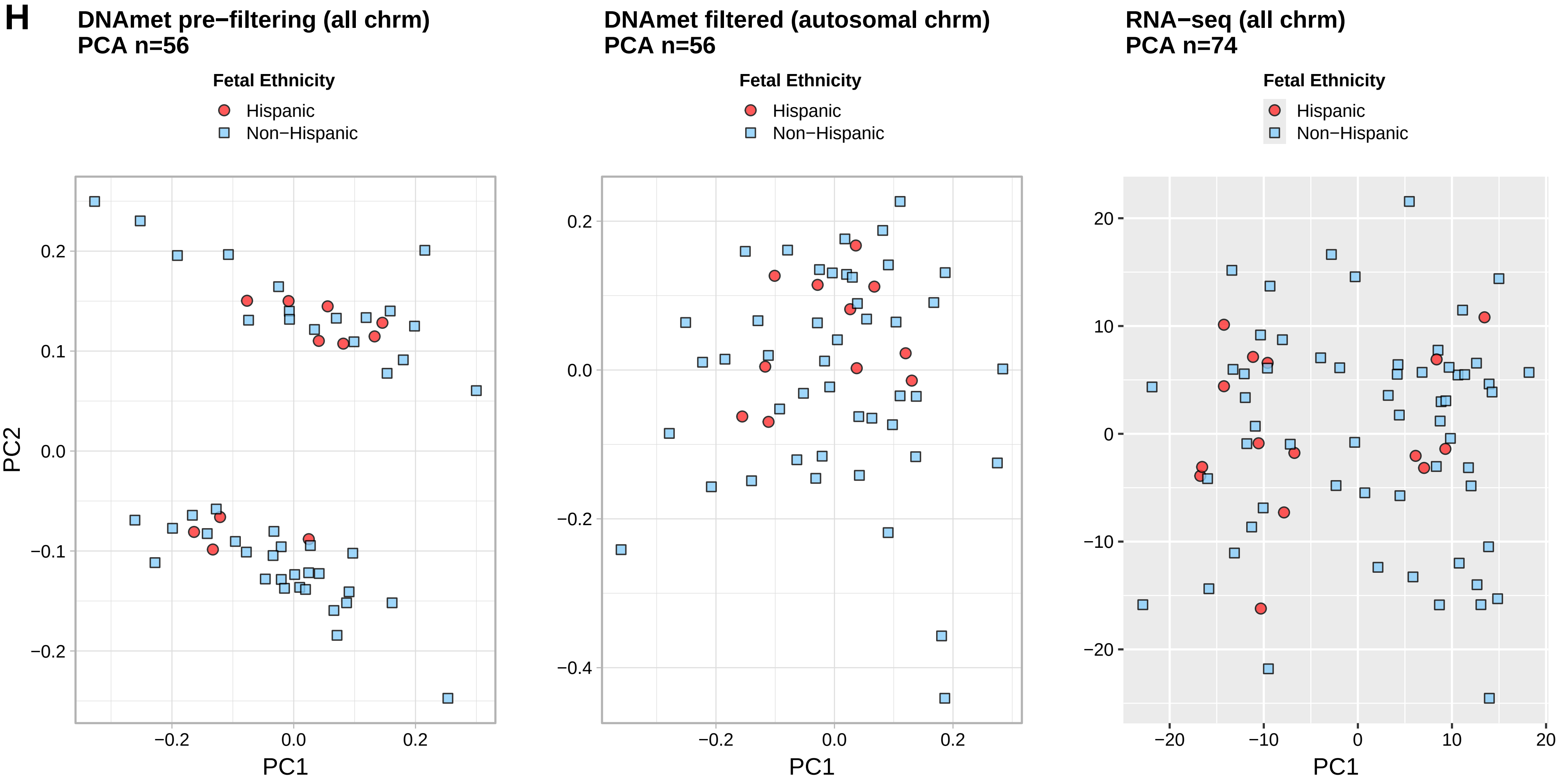
H
DNAmet pre−filtering (all chrm)
PCA n=56
Fetal Ethnicity
Hispanic
Non−Hispanic
PC2
PC1
DNAmet filtered (autosomal chrm)
PCA n=56
Fetal Ethnicity
Hispanic
Non−Hispanic
PC1
RNA−seq (all chrm)
PCA n=74
Fetal Ethnicity
Hispanic
Non−Hispanic
PC1

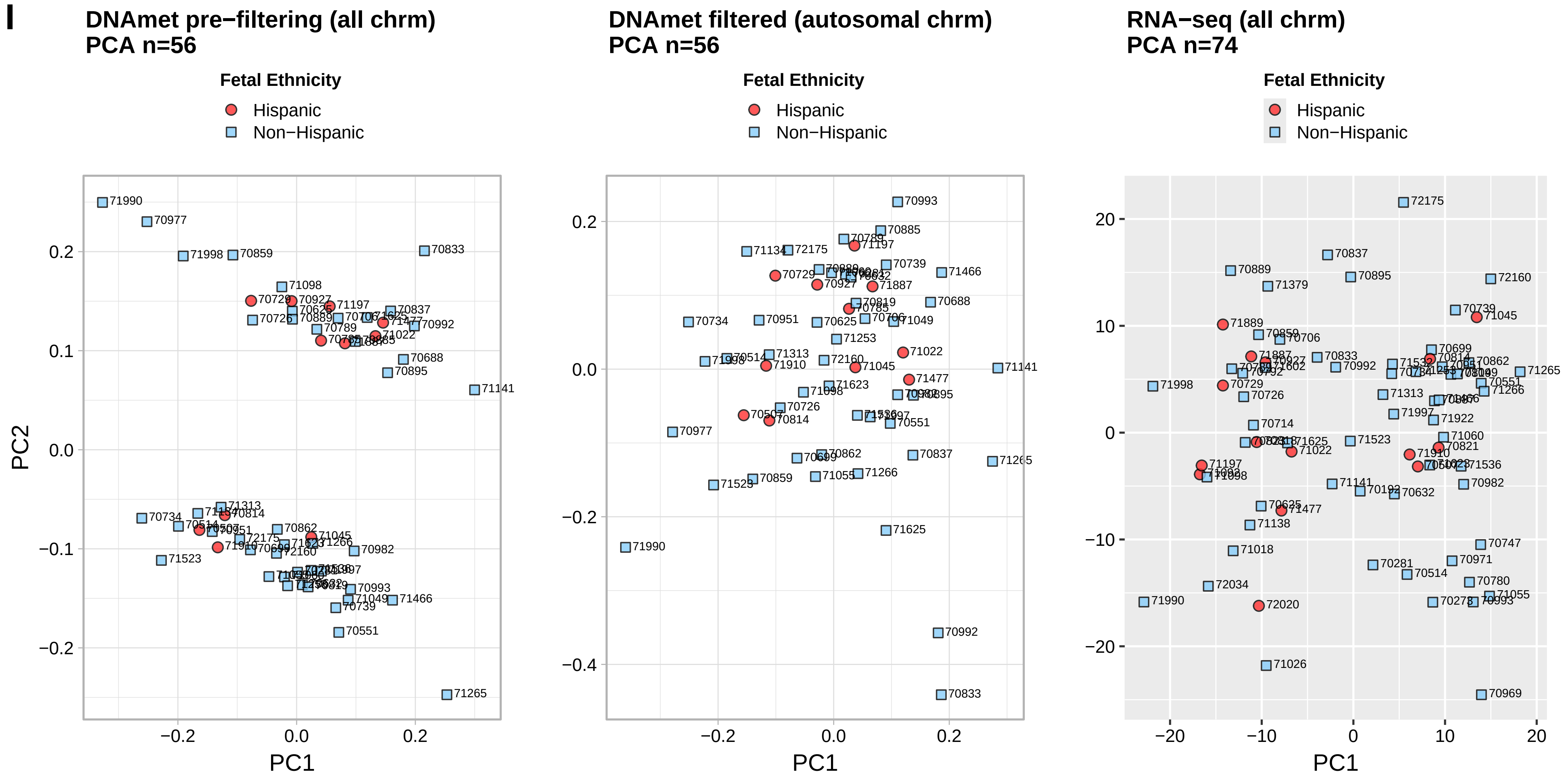
I
DNAmet pre−filtering (all chrm)
PCA n=56
Fetal Ethnicity
Hispanic
Non−Hispanic
PC2
PC1
DNAmet filtered (autosomal chrm)
PCA n=56
Fetal Ethnicity
Hispanic
Non−Hispanic
PC1
RNA−seq (all chrm)
PCA n=74
Fetal Ethnicity
Hispanic
Non−Hispanic
PC1

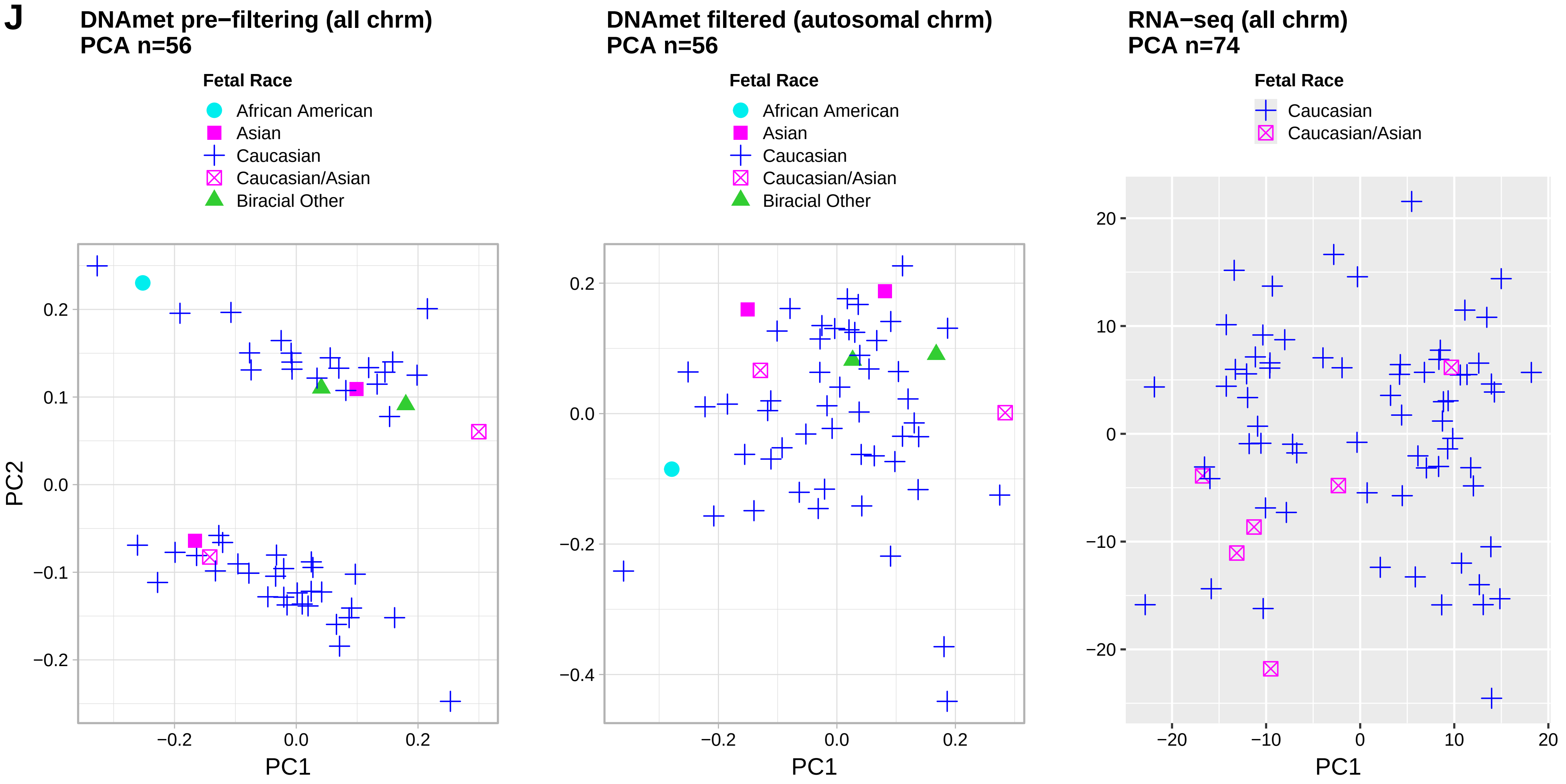
J
DNAmet pre−filtering (all chrm)
PCA n=56
Fetal Race
African American
Asian
Caucasian
Caucasian/Asian
Biracial Other
PC2
PC1
DNAmet filtered (autosomal chrm)
PCA n=56
Fetal Race
African American
Asian
Caucasian
Caucasian/Asian
Biracial Other
PC1
RNA−seq (all chrm)
PCA n=74
Fetal Race
Caucasian
Caucasian/Asian
PC1
